# Application of third-generation sequencing to herbal genomics

**DOI:** 10.3389/fpls.2023.1124536

**Published:** 2023-03-07

**Authors:** Longlong Gao, Wenjie Xu, Tianyi Xin, Jingyuan Song

**Affiliations:** Key Lab of Chinese Medicine Resources Conservation, State Administration of Traditional Chinese Medicine of the People’s Republic of China, Engineering Research Center of Chinese Medicine Resource of Ministry of Education, Institute of Medicinal Plant Development, Peking Union Medical College and Chinese Academy of Medical Sciences, Beijing, China

**Keywords:** third-generation sequencing, PacBio, nanopore, herbal genomics, medicinal plant, molecular identification

## Abstract

There is a long history of traditional medicine use. However, little genetic information is available for the plants used in traditional medicine, which limits the exploitation of these natural resources. Third-generation sequencing (TGS) techniques have made it possible to gather invaluable genetic information and develop herbal genomics. In this review, we introduce two main TGS techniques, PacBio SMRT technology and Oxford Nanopore technology, and compare the two techniques against Illumina, the predominant next-generation sequencing technique. In addition, we summarize the nuclear and organelle genome assemblies of commonly used medicinal plants, choose several examples from genomics, transcriptomics, and molecular identification studies to dissect the specific processes and summarize the advantages and disadvantages of the two TGS techniques when applied to medicinal organisms. Finally, we describe how we expect that TGS techniques will be widely utilized to assemble telomere-to-telomere (T2T) genomes and in epigenomics research involving medicinal plants.

## 1 Introduction

There is a long history of traditional medicine use. In 2015, Chinese scientist Youyou Tu won the Nobel Prize for her outstanding contribution to the discovery of artemisinin, refreshing the global perception of traditional Chinese medicine. There is little doubt that many further health-promoting discoveries will be made by studying traditional medicine. Over recent decades, many phytochemical and pharmacological research projects have investigated the bioactive components and underlying mechanisms of herbal medicine. However, the available genetic information on herbal medicines long remained lacking due to the high cost of the predominant first-generation sequencing technique, Sanger sequencing. It was not until the emergence of next-generation sequencing (NGS) that the situation improved. Because of its high-throughput and low costs, NGS has made it affordable for most researchers to sequence the genomes and transcriptomes of medicinal plants, greatly promoting the development of herbal genomics. Nevertheless, as research developed, the inherent shortcomings of NGS—especially the short read lengths—became a new bottleneck hindering the development of herbal genomics.

When using NGS techniques, the characteristics of short read lengths make it difficult to assemble the raw fragments into high-quality contigs or scaffolds, especially those with high heterozygosity or a high proportion of repeat sequences. Yet, the development of third-generation sequencing (TGS) has brought us a great opportunity to solve these problems. However, TGS is still unfamiliar to many researchers. Therefore, here we introduce the principles, pipelines, and sequencing instruments of two mainstream TGS techniques, PacBio single-molecule real-time (SMRT) sequencing technology and Oxford Nanopore technology (ONT). We compare these two techniques with Illumina, the predominant NGS technique. To demonstrate how TGS can be applied to herbal genomics, we have chosen several classic studies of genomics, transcriptomics, and molecular identification as examples to dissect the specific processes and summarize the advantages and disadvantages of TGS when applied in medicinal organisms. This work will provide a meaningful reference for traditional medicine and genomic researchers.

## 2 Insights into main TGS

The first single-molecule sequencing technology was developed by Helicos Bioscience, but it is rarely used now because it is comparatively time-consuming and has short read lengths (~32 bp) (Harris et al., 2008; Orlando et al., 2011). Currently, there are two widely used TGS technologies, PacBio SMRT technology and ONT.

### 2.1 PacBio SMRT technology

#### 2.1.1 The principle of SMRT

**Figure 1:**
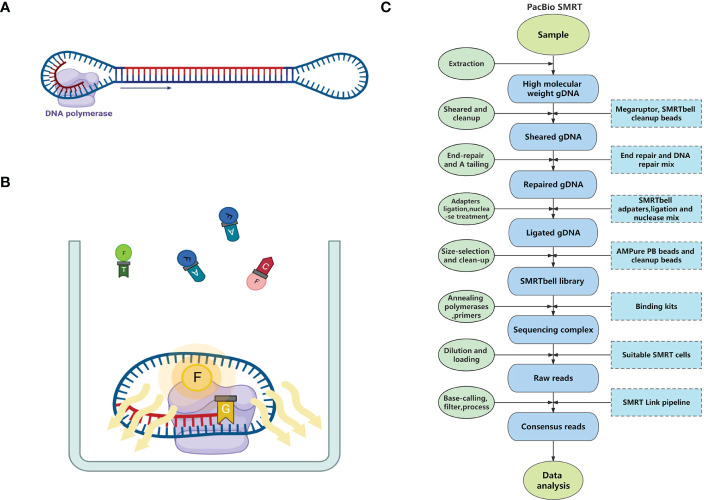
The principle and pipeline of PacBio SMRT Technology. **(A)** A dumbbell-shaped template for sequencing (SMRTbell) consists of adapters in both ends, a double-stranded DNA template, and a DNA polymerase. **(B)** The process of sequencing in the Zero-model waveguide (ZMW). **(C)** The pipeline of PacBio SMRT sequencing.

Similar to Illumina sequencing, SMRT is based on the principle of sequencing-by-synthesis, acquiring sequence information during the amplification process of nucleic acid molecules. Before sequencing, both ends of the targeted double-stranded DNA (dsDNA) molecule are ligated with hairpin adapters to form dumbbell-shaped templates (i.e., SMRTbells). These adapters allow DNA polymerases and primers to bind with the SMRTbells (**Figure 1A**). After binding, the SMRTbells are sequenced on a SMRT cell chip. There are thousands of Zero-model waveguides (ZMWs) lined up on each SMRT cell. The ZMWs limit the observation volume to avoid the influence of the fluorescence of uncombined deoxyribonucleoside triphosphates (dNTPs), which allows the detection of a single dNTP. Once the SMRTbells are loaded onto the SMRT cell, a proportion of them fall into the ZMWs. Then, the SMRTbells are fixed on the bottom of ZMWs through the interaction between the biotin on the polymerase and the streptavidin on the glass plate of the ZMWs (Eid et al., 2009). The DNA polymerases catalyze the continuous incorporation of dNTPs labeled by different fluorophores into complementary strands (Flusberg et al., 2010). When the polymerases capture the labeled dNTPs, they emit distinct fluorescence pulses under excitation light (**Figure 1B**). Four classic dNTP types can be recognized from their featured fluorescence signature (Eid et al., 2009).

The methylated bases in the DNA template change the incorporation kinetics of polymerases, which enables SMRT to directly detect methylated bases without chemical modifications (Flusberg et al., 2010). Moreover, because of the circular structure of the SMRTbell and the replacement sequencing ability of the DNA polymerase, inserted DNA templates can be repeatedly sequenced, yielding many copies of both template and complementary strands. Aligning these copies greatly improves the sequencing accuracy. This sequencing strategy is also known as circular consensus sequencing (CCS) (Travers et al., 2010).

#### 2.1.2 The pipeline of SMRT

Sample and library preparation: In the genome sequencing of medicinal plants, fresh leaves are often used as samples for DNA extraction. High molecular weight genomic DNA (HMW gDNA) should be extracted from the samples because the amount and length distribution of extracted gDNA are important for subsequent library construction. Usually, high fidelity (HiFi) library construction for whole genome sequencing of plants requires at least 1 μg of DNA input per 1 Gb of genome length. DNA molecules ≥10 kb should account for more than 90%, and molecules ≥30 kb should account for more than 50%. Additionally, when this amount of DNA cannot be extracted from the samples, alternative workflows are available (low DNA input workflow and ultra-low DNA input workflow). After quality control of HMW gDNA molecules, they need to be sheared to suitable sizes by the Megaruptor system, followed by cleanup with SMRTbell cleanup beads. Then, the sheared gDNA undergoes end-repair, A tailing, adapters ligation, and nuclease treatment in a thermocycler, followed by size-selection using AMPure PB Beads or cleanup with SMRTbell cleanup beads to form the SMRTbell library (usually 15–18 kb). Finally, DNA polymerases and primers are annealed to the SMRTbell library using Binding kits (e.g., Sequel II binding kits 3.2), and then the final sequencing complexes are constructed^1^.

Sequencing: After dilution, the SMRTbell library is loaded onto PacBio sequencers with one or more SMRT cells, each of which can yield HiFi reads up to 4 Gb in one run. The runtime is flexible depending on the amount of data needed by the assembly^2^.

Primary data analysis: Base-calling and primary filtering analysis are performed on the sequencer. SMRT Link, PacBio DevNet, and other software tools are available to process the raw SMRT data^3^. The complete pipeline of SMRT is shown in **Figure 1C**.

#### 2.1.3 Sequencing instruments of SMRT

There are six long-read sequencing instruments based on SMRT sequencing technology: PacBio RS, RSII, sequel, sequel II, sequel IIe, and the newly released Revio. Among them, PacBio RS is the first sequencer commercialized by PacBio. As an early-released instrument, PacBio RS had a relatively low throughput, short average read lengths (~1.5 kb) and a high error rate (~13%) (Quail et al., 2012). With technological advances, the throughput of PacBio sequencers has increased by several hundred folds while the average read lengths underwent a 10-fold increase. The accuracy has also been improved to more than 99% due to the extensive use of the CCS strategy (**Table 1**). These instruments have been used in many genomic and transcriptomic studies of medicinal plants, among which PacBio sequel is used frequently, probably because of its high throughput and relatively low costs.

**Table 1:** Comparison of SMRT, ONT and Illumina representative instruments.

Technique	Instrument	Principle	Accuracy	Read length	Throughput per run/Gb	Run time/h	Reference
**PacBio SMRT**	RS	Sequencing-by-synthesis	~87%	~1.5 kb	0.1	2	(Quail et al., 2012)
	Sequel II		≥99%	~15-18 kb	30	30	PacBio website^10, 11^,
	Revio				360	24	
**Oxford Nanopore**	MinION	Threading DNA or RNA through nanopore protein	~85%	Equal to the length of input DNA or RNA	50	72	Nanopore website ^12, 13^, (Jain et al., 2017)
	GridION				250	72	
	PromethION				14,000	72	
**Illumina**	Miniseq	Sequencing-by-synthesis	~99.6%	2×150 bp	7.5	4-24	Illumina website^14^ (Quail et al., 2012)
	Miseq			2×300 bp	15	4-55	
	Nova-Seq X			2×150 bp	16,000	13-48	

10 1^0^ https://www.pacb.com/technology/hifi-sequencing/sequel-system,11 1^1^ https://www.pacb.com/revio/12 1^2^ https://nanoporetech.com/products13 1^3^ https://nanoporetech.com/products/kits14 1^4^ https://www.illumina.com.cn/systems/sequencing-platforms.html

### 2.2 Oxford nanopore technology

ONT is another popular TGS technique. Unlike previous sequencing technologies, ONT does not detect fluorescence, light, or pH signals. Instead, it distinguishes bases by detecting electrical signals (Clarke et al., 2009).

#### 2.2.1 The principle of ONT

**Figure 2:**
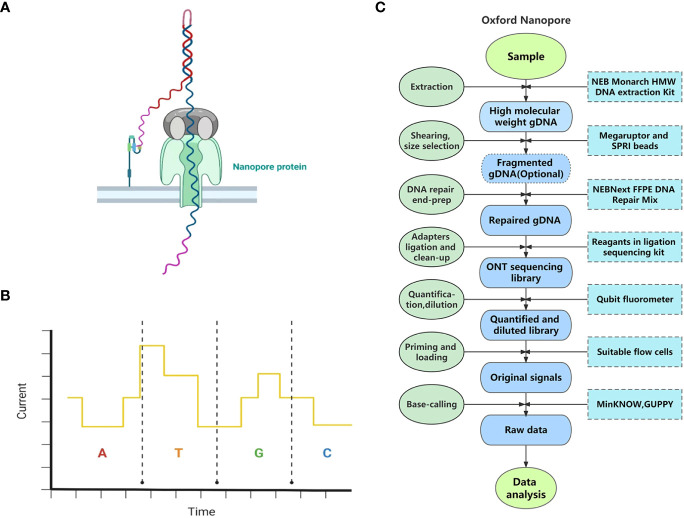
The principle and pipeline of Oxford Nanopore Technology. **(A)** The specific scene of threading a single-stranded DNA through a nanopore protein. **(B)** Different dNTPs can cause distinct interference to the current when passing through the nanopore protein (the current variations demonstrated do not represent the true influence of dNTPs.) **(C)** The pipeline of Oxford Nanopore Technology.

ONT originated from a brand-new idea of threading a single-stranded nucleic acid molecule through a nanopore protein (Clarke et al., 2009) (**Figure 2A**). The sequencing process of ONT takes place in a container filled with an electrolyte solution. A lipid double-layer membrane embedded with a nanopore protein is placed in the container. Under an applied voltage, a stable current is formed in the nanopore due to the flow of ions. Therefore, when a nucleic acid molecule passes through, the nanopore is partially blocked, and the stable current is interfered with. Because the structures of nucleotides are different, they cause distinct interferences with the current (**Figure 2B**). For this reason, nucleotides of sequenced nucleic acid molecules can be distinguished from their distinctive current variations; in this way, sequence information can be decoded (Clarke et al., 2009; Jain et al., 2016).

#### 2.2.2 The pipeline of ONT

Sample and library preparation: Library preparation kits for whole genome sequencing, targeted DNA sequencing, and RNA sequencing are all provided by ONT^4^. Here we take genome sequencing on MinION using Ligation Sequencing Kit V14 as an example. The process of library preparation using this sequencing kit takes about 60 min. Fresh leaves are frequently used for DNA extraction in whole genome sequencing by ONT. First, HMW gDNA can be extracted from plant tissues using the NEB Monarch HMW DNA Extraction Kit or other compatible extraction kits. Then, researchers can choose whether to conduct fragmentation or size selection in the pipeline. If not, the yielded read length will equal the input fragment length. Second, the extracted HMW gDNA undergoes DNA repair and end-preparation (end-prep) using NEBNext FFPE DNA Repair Mix and NEBNext Ultra II End Repair/dA-tailing Module reagents. Third, sequencing adapters are ligated to the repaired ends of DNA molecules using a ligation sequencing kit and some other reagents, followed by cleanup, after which the sequencing library is prepared. Finally, about 1 μl of the DNA library is quantified using a Qubit fluorometer, and then the DNA library is produced as 12 µl at 10–20 fmol^5^. In addition, an automated device, VolTRAX, released by ONT enables hands-free and standard sequencing library construction^6^.

Sequencing: Different library preparation kits are compatible with different versions of flow cells, which should be confirmed before sequencing. After priming the flow cell, 10–20 fmol of the final DNA library is advised to be loaded onto the flow cell. The time for one run is up to 72 h.

Primary data analysis: Data acquisition is usually performed by MinKNOW, and base-calling can be conducted by MinKNOW, GUPPY, and many other algorithms available on GitHub^7^. The complete pipeline of ONT is shown in **Figure 2C**.

#### 2.2.3 Sequencing instruments of ONT

Flongle, MinION, GridION, and PromethION are the main sequencing instruments of ONT. Among them, GridION and PromethION are bench-top instruments with high throughput and, therefore, usually used for large-scale sequencing projects^8^ (Jain et al., 2016) such as whole genome sequencing of humans, mammal animals, and plants. MinION is a portable instrument weighing only 90 g that can be used for small sequencing projects, such as microorganism genomes and rapid sequencing outside the laboratory (and even in space) (Jain et al., 2016; McIntyre et al., 2016). Flongle is a single-use product generating 1–2 Gb of data, which is suitable for even smaller projects, such as plasmid and viral sequencings^9^.

### 2.3 Comparison between TGS and NGS techniques

There are three main strengths of SMRT and ONT compared to Illumina, the predominant NGS technique (**Table 1**). First, the average read length of SMRT and ONT (usually ≥10 kb) is much longer than that of Illumina (~150–300 bp). Second, SMRT and ONT have much lower guanine and cytosine-content bias (GC bias) than Illumina and other NGS techniques (Benjamini and Speed, 2012; Roeh et al., 2017; Sato et al., 2019; Castaño et al., 2020). Third, SMRT and ONT enable researchers to directly detect base modifications without any of the special processes needed by Illumina (Flusberg et al., 2010; Simpson et al., 2017; LaBarre et al., 2019). However, it is worth noting that although the accuracy of SMRT can be greatly improved by the CCS strategy (Travers et al., 2010), the error rate of ONT (~15%) (Jain et al., 2017) is still much higher than that of Illumina (~0.4%) (Quail et al., 2012).

### 2.4 Comparison between ONT and SMRT

Both ONT and SMRT are single-molecule sequencing techniques with long read lengths, low GC bias, and the ability to directly detect base modifications. However, there are many differences between these two techniques. First, the principles of ONT and SMRT are very different. SMRT inherited and developed the basic principle and labeling method of Illumina sequencing, namely sequencing-by-synthesis and fluorescence labeling, while ONT is a novel approach that threads the nucleic acid molecules through nanopore proteins and distinguishes nucleotides by electrical signals. Second, the types of instruments of SMRT and ONT are different. Sequencers of SMRT are all bench-top instruments with relatively high throughput, while ONT devices can be either bench-top, high-throughput devices (GridION, PromethION) or portable, comparatively low-throughput devices (MinION, Flongle) are available (**Table 1**).

## 3 Application of TGS to herbal genomics

### 3.1 Decoding whole genomes of medicinal plants

#### 3.1.1 Nuclear genomes

**Figure 3:**
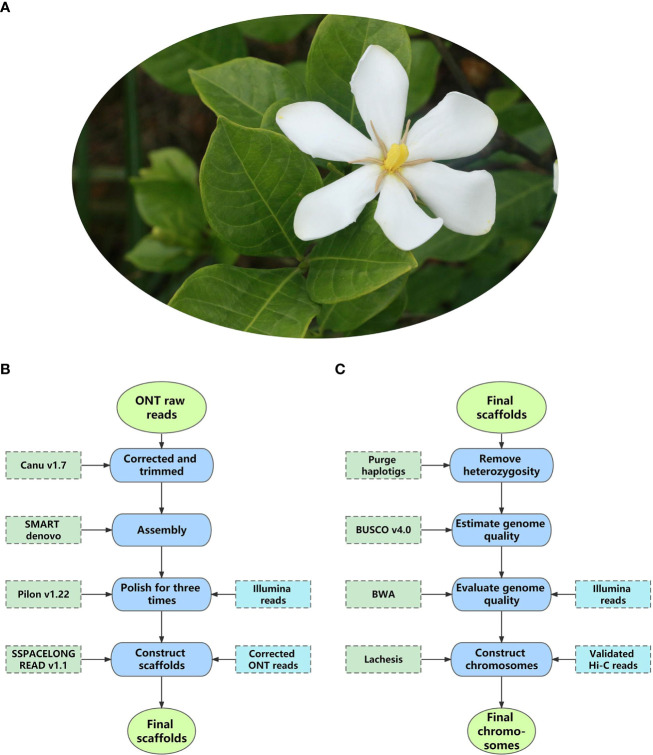
The pipeline of chromosome-level assembly of *Gardenia jasminoides* genome. **(A)** The flower of *Gardenia jasminoides*. **(B)** The pipeline for assembling the raw data into scaffolds. **(C)** The pipeline for assembling scaffolds into pseudo-chromosomes.

Decoding nuclear genomes of medicinal plants with high heterozygosity and a high proportion of repeat sequences using NGS techniques often leads to fragmented genome assemblies. With much longer read lengths, TGS enables researchers to uncover the genomic regions missed by NGS techniques. To date, more than 100 nuclear genomes of medicinal plants have been sequenced using TGS, most of which are assembled to a chromosome level combined with high-throughput chromosome conformation capture (Hi-C) mapping technology (Cheng et al., 2021c) (**Supplementary Table 1**). Among them, a recent study of *Gardenia jasminoides* (**Figure 3A**) demonstrated the classic processes of applying TGS to nuclear genomes research of medicinal plants, from library construction to data analysis. Therefore, it is taken as an example here (Xu et al., 2020b).

In the reference study, ONT, Illumina, and Hi-C are combined to gain a chromosome-level assembly of the *G. jasminoides* genome, while RNA sequencing (RNA-seq) is used to evaluate assembly quality, predict protein-coding genes, and calculate the expression level of genes.

Genome size estimation: Before ONT sequencing, flow cytometry (Pfosser et al., 1995) and *k*-mer distribution analysis (Manekar and Sathe, 2018) were used to estimate genome size and heterozygosity. Based on the two methods, the genome of *Gar. jasminoides* was predicted to have a total size of 550.6 ± 9 Mb and a high heterozygosity of 2.2%, implicating that it is challenging to assemble this genome.

Library preparation: Libraries for ONT, Illumina, RNA-seq, and Hi-C were constructed. In the reference study, the fresh leaves of *Gar. jasminoides* were pooled for DNA extraction of ONT and Illumina sequencing. Seven organs, including fruits at different maturity stages, of *Gar. jasminoides* were collected for RNA-seq and the measurement of crocin content. The Hi-C library was constructed with fresh tissue from *Gar. jasminoides*. For ONT library construction, HMW gDNA was extracted from the pooled leaves and then fragmented, size selected, and purified to get large fragments, after which the large fragments underwent end-prep, adapter ligation, tether attachment, and then an ONT library was constructed.

Sequencing and assembly: The complete ONT library of *Gar. jasminoides* was sequenced on GridION X5, and the raw data was base-called using Guppy (v1.8.5), generating 2.67 Gb reads with an N50 of 21.6 kb. For assembly, the authors developed a satisfactory package (Canu-SMARTdenovo-3×Pilon) by testing various *de novo* assembly pipelines. Specifically, using this package, base-called ONT reads were corrected and trimmed by Canu and then assembled with SMARTdenovo, followed by Illumina short reads to polish the Canu-SMARTdenovo contigs with Pilon three times. The final scaffolds were assembled with the polished contigs, and ONT reads were corrected using Canu; heterozygous sequences were eliminated by Purge Haplotigs. Finally, the researchers acquired a 534.1 Mb assembly with a contig N50 of 1.0 Mb (**Figure 3B**). The quality of the assembly was evaluated using Benchmarking Universal Single-Copy Orthologs (BUSCO) and mapped with Illumina short reads from the DNA and RNA libraries of *Gar. jasminoides*, respectively, which found 95.0% complete BUSCOs. Hi-C technology was used to further improve the quality of the assembly. As a result, 99.5% of sequences from the assembly were scaffolded into 11 pseudo-chromosomes using the Lachesis package. At this stage, the final chromosome-level genome of *Gar. jasminoides* was successfully constructed. It was 535 Mb in size, with a scaffold N50 of 44 Mb (**Figure 3C**).

**Figure 4:**
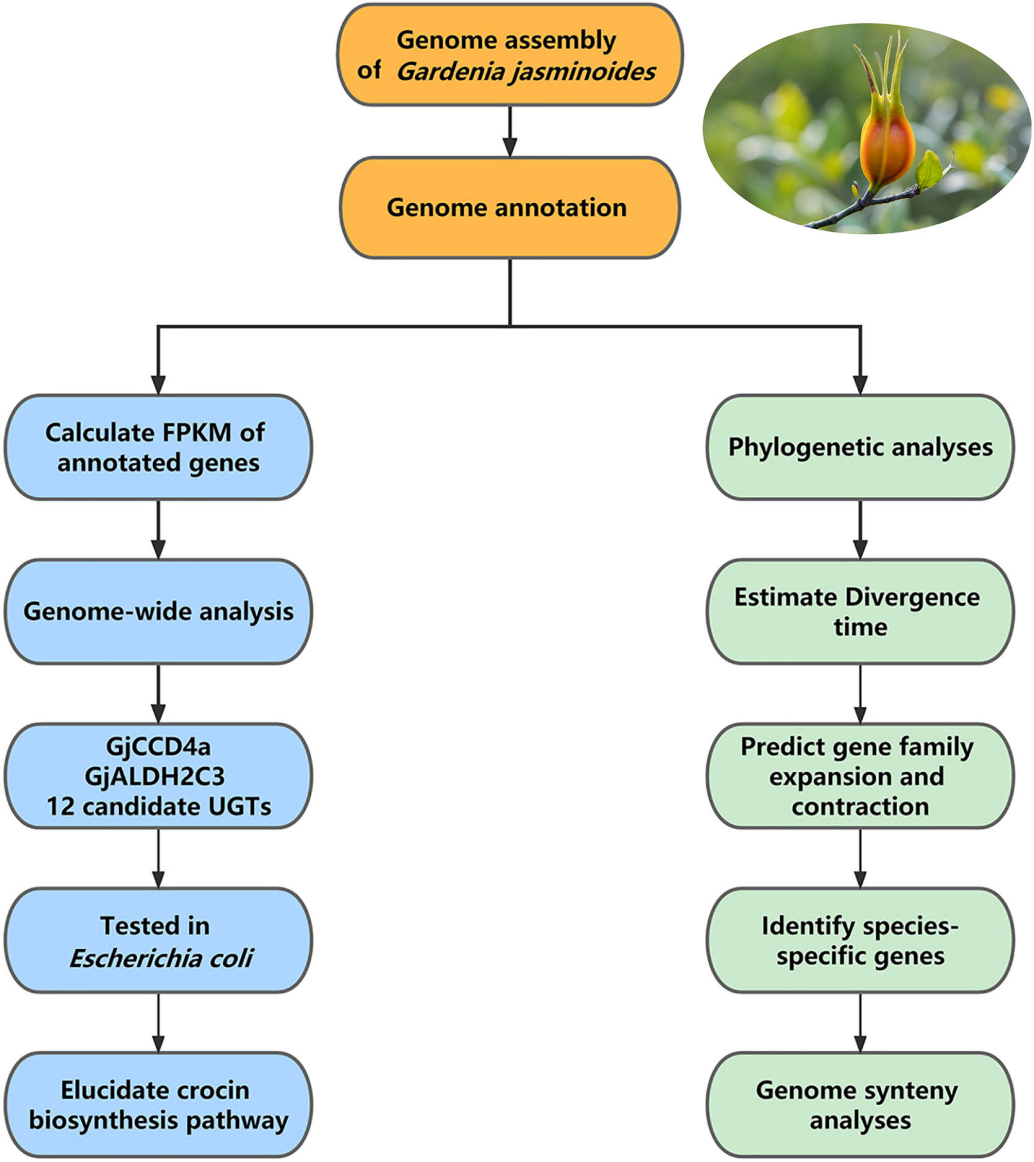
The workflow of data analysis of *Gardenia jasminoides* genome. The data analysis of the *Gardenia jasminoides* genome mainly includes genome annotation followed by comparative analysis and expression analysis of annotated genes.

Further data analysis: The first step of data analysis for medicinal plant genomes is usually genome annotation, followed by comparative genomic analysis and expression analysis. In the reference study, at first, the chromosome-level genome of *Gar. jasminoides* was used for genome annotation, including the annotation of repeat elements, the prediction and functional annotation of protein-coding genes, and the annotation of non-coding RNA. This genome was then used for comparative genomic analyses, including synteny analysis between *Gar. jasminoides* and *Coffea canephora*, phylogenetic analysis between *Gar. jasminoides* and ten additional angiosperms, followed by mapping transcriptome reads obtained from RNA-seq of seven organs of *Gar. jasminoides* to the annotated genes to calculate the relative expression level of genes (fragments per kilobase of exon per million reads mapped, FPKM). Furthermore, genome-wide analysis was conducted after mapping, in which genes from three families related to crocin biosynthesis were identified, including 14 carotenoid cleavage dioxygenases (CCDs) genes, 18 aldehyde dehydrogenases (ALDHs)-like genes, and 237 UDP-glucosyltransferases (UGTs) genes. The *Gar. jasminoides* crocin biosynthetic was elucidated after expressing 14 candidate crocin biosynthetic genes in *Escherichia coli* to test their enzymatic activity. In addition, the above-mentioned comparative analysis revealed the evolution of crocin and caffeine biosynthesis genes in Rubiaceae (**Figure 4**).

In addition to the software used in the reference study, many new tools suitable for TGS techniques have recently been developed. Ratatosk (Holley et al., 2021) was developed for hybrid error correction while CONSENT (Morisse et al., 2021) was designed for self-correction of TGS. For genome assembly, new assemblers are available, such as hifiasm (Cheng et al., 2021a) and Nextdenovo (https://github.com/Nextomics/NextDenovo). And for scaffolding, RegScaf (Li and Li, 2022) was developed to resolve large genomes and repeat regions, while YaHS (Zhou et al., 2023) is suitable for chromosome-scale scaffold construction using Hi-C data. Regarding annotation, PhyloCSF++ (Pockrandt et al., 2022) is a newly updated tool for differentiating protein-coding and non-coding regions, and TransposonUltimate (Riehl et al., 2022) is a newly developed tool for transposon classification, annotation, and detection. Advanced bioinformatics software have also greatly facilitated the genome research of medicinal plants.

High-quality reference genome assemblies have provided valuable genetic information for investigating the biosynthesis of secondary metabolites, such as triptolide, morphinan, and icaritin. By integrating the genome, transcriptome, and metabolome of *Tripterygium wilfordii*, a cytochrome P450 (CYP728B70) in *T. wilfordii* was identified to catalyze the oxidation of a methyl to the acid moiety of dehydroabietic acid in triptolide biosynthesis, providing clues for elucidating the biosynthetic pathway of triptolide (Tu et al., 2020). *(S)- to (R)-reticuline* (*STORR*) gene fusion is key for morphinan biosynthesis in *Papaver somniferum*. CYP450 and oxidoreductase genes that combined to form the gene fusion were also identified by paralog analysis using a chromosome-level *Papaver somniferum* genome assembly (Guo et al., 2018). In addition, an important flavonoid prenyltransferase (*Ep*PT8) in *Epimedium pubescens* was proven to be involved in the biosynthesis of icaritin and its derivatives by whole genome search using a chromosome-level genome assembly of *E. pubescens* (Shen et al., 2022a). Notably, the biosynthetic pathways of iridoids in *Rehmannia glutinosa* (Ma et al., 2021) and crocin in *Gardenia jasminoides* (Xu et al., 2020b) were successfully elucidated.

The availability of high-quality reference genomes also facilitates the research of the evolutionary history of important gene clusters, biosynthetic pathways, and species in different families. For example, the convergent evolution of CYP82D and CYP706X members in Lamiaceae and Asteraceae (Gao et al., 2022) and the divergent evolution of caffeine and crocin biosynthetic pathways were revealed based on TGS genome assemblies. Information on the evolution of species in Euphorbiaceae (Wang et al., 2021b), Asteraceae (Shen et al., 2018), and Magnoliid (Shang et al., 2020) has been provided as well in recent studies. These are valuable resources for subsequent functional genomics, molecule-assisted breeding, and synthetic biology research (Xin et al., 2019).

Although many chromosome-level assemblies of medicinal plant genomes have been completed, gaps and highly repetitive regions remain to be resolved, such as the centromere and telomere regions. Recently, by combining ONT ultra-long reads, PacBio HiFi reads, and Hi-C technology, researchers successfully obtained truly gapless telomere to telomere (T2T) reference genome assemblies of *Hordeum vulgare* (Navrátilová et al., 2022), *Arabidopsis thaliana* (Wang et al., 2022a), *Citrullus lanatus* (Deng et al., 2022), and several other plants. Compared to chromosome-level assemblies, T2T assemblies are more complete, can be used to discover almost all genomic variations, and enable research into centromere and telomere regions. However, T2T assembly of medicinal plants is still rarely reported.

#### 3.1.2 Organelle genomes

Organelle genomes are also important genetic resources for medicinal plant utilization. To date, the chloroplast genomes (cp-genomes) of more than 20 important medicinal plants (**Table 2**) have been sequenced using TGS. Further, the complete mitochondrial genomes of several important medicinal plants have been sequenced by TGS (**Table 3**). Most of the obtained cp-genomes were determined as circular molecules with quadripartite structures consisting of a pair of inverted repeat regions (IRs), a large single-copy region (LSC), and a small single-copy region (SSC), while a few of which were also identified as tripartite or bipartite structures. Here we take the research of *Salvia miltiorrhiza* (Chen et al., 2014) as an example to introduce the achievements of this research and the classic workflow when applying TGS to the sequencing of medicinal plant cp-genomes.

**Table 2:** Chloroplast genome assemblies obtained using TGS techniques.

Family	Species	Technique	Total size/kb	Structure	Protein-coding genes	rRNA	tRNA	GC	Reference
Dicotyledonae									
Aristolochiaceae	*Asarum heterotropoides*	O,I	190.2	Tri	100	8	38	36.78%	(Yoo et al., 2021)
	*Asarum maculatum*	O,I	193.1	Tri	100	8	38	36.24%	(Yoo et al., 2021)
	*Asarum misandrum*	O,I	193.2	Tri	99	8	38	36.22%	(Yoo et al., 2021)
Asteraceae	*Carthamus tinctorius*	P	153.0	Qua	79 (Uni)	4 (Uni)	29 (Uni)	37.80%	(Wu et al., 2019)
	*Chrysanthemum boreale*	P	151.0	Qua	87	8	46	37.47%	(Won et al., 2018)
Cucurbitaceae	*Luffa acutangula*	P,I	157.2	Qua	87	8	36	37.14%	(Yundaeng et al., 2020)
	*Luffa aegyptiaca*	P,I	157.3	Qua	87	8	36	37.12%	(Yundaeng et al., 2020)
Fabaceae	*Callerya reticulata*	O,I	132.5	Bi	74	4	30	34.19%	(Chen et al., 2022b)
	*Callerya nitida*	O,I	132.4	Bi	74	4	30	33.89%	(Chen et al., 2022b)
Lamiaceae	*Salvia miltiorrhiza*	P,I	151.3	Qua	86	8	37	38%	(Qian et al., 2013)
Ranunculaceae	*Aconitum barbatum* var. *puberulum*	P,S	156.7	Qua	84	34	8	38.7%	(Chen et al., 2015)
Gentianaceae	*Swertia mussotii*	P	153.4	Qua	84	8	37	38.20%	(Xiang et al., 2016)
Loranthaceae	*Taxillus chinensis*	P,I	121.4	Qua	66	8	28	37.3%	(Li et al., 2017b)
Loranthaceae	*Taxillus sutchuenensis*	P,I	122.6	Qua	66	8	28	37.3%	(Li et al., 2017b)
Caricaceae	*Vasconcellea pubescens*	O,I	158.7	Qua	82	8	37	37%	(Lin et al., 2020)
Nelumbonaceae	*Nelumbo nucifera*	P,I	163.6	Qua	85	8	37	**/**	(Wu et al., 2014)
Monocotyledoneae									
Liliaceae	*Fritillaria hupehensis*	P	152.1	Qua	89	8	38	36.97%	(Li et al., 2014)
	*Fritillaria taipaiensis*	P	151.7	Qua	89	8	38	36.97%	(Li et al., 2014)
	*Fritillaria cirrhosa*	P	152.0	Qua	89	8	38	36.95%	(Li et al., 2014)
	*Fritillaria unibracteata* var. *wabuensis*	P	151.0	Qua	88	8	37	37.0%	(Li et al., 2016)
	*Lilium rosthornii*	P,I	152.2	Qua	85	8	38	37.02%	(Wu et al., 2021a)
Poaceae	*Coix lacryma-jobi*	P,I	140.9	Qua	87 (Uni)	4 (Uni)	32 (Uni)	**/**	(Kang et al., 2018)
Zingiberaceae	*Curcuma longa*	P,I	162.2	Qua	87	8	36	36.20%	(Li et al., 2019)
Araceae	*Spirodela polyrhiza*	P	169.0	Qua	85	8	36	35.68%	(Zhang et al., 2020b)
Orchidaceae	*Dendrobium officinale*	P,I	152.2	Qua	89	8	30	37.46%	(Zhong et al., 2016)

P, PacBio SMRT; O, Oxford Nanopore; I, Illumina; S, Sanger;/, not reported; Tri, Tripartite; Qua, Quadripartite; Bi, Bipartite; Uni, the number of unique genes.

**Table 3:** Mitochondrial genome assemblies obtained using TGS techniques.

Family	Species	Tech-nique	Total size/kb	GC content	Protein-coding genes	tRNA	rRNA	Repeat sequence	Reference
Magnoliaceae	*Magnolia biondii*	O	967.1	46.6%	41	20	3	27%	(Dong et al., 2020)
Lamiaceae	*Scutellaria tsinyunensis* Conformation A	O,I,S	354.1	45.26%	32	24	3	**/**	(Li et al., 2021b)
	*Scutellaria tsinyunensis* Conformation B	O,I,S	255.7, 98.4	45.26%	32	24	3	**/**	(Li et al., 2021b)
Fabaceae	*Dalbergia odorifera*	P,I	435	45.1%	33	17	4	4.0%	(Hong et al., 2021)
Fabaceae	*Sophora japonica*	P,I	484.916	45.4%	32	17	3	4%	(Shi et al., 2018)
Umbelliferae	*Coriandrum sativum* circle 1	O,I	82.926	**/**	14	12	2	**/**	(Wang et al., 2021c)
Umbelliferae	*Coriandrum sativum* circle 2	O,I	224.59	**/**	41	16	5	**/**	(Wang et al., 2021c)

P, PacBio SMRT; O, Oxford Nanopore; I, Illumina; S, Sanger;/, not reported.

Sample and library preparation: The sample and library preparation of cp-genomes is similar to that of nuclear genomes. In the selected study, fresh leaves were prepared from *S. miltiorrhiza* for gDNA isolation. The gDNA was extracted using a plant genomic DNA kit (Tiangen, China). Libraries consisting of inserted fragments 1 kb and 10 kb in size were prepared and used for subsequent SMRT sequencing.

Sequencing and assembly: The SMRT sequencing of gDNA was conducted under the guidance of the manufacturer provided by PacBio and the raw sequences were preprocessed using the SMRT Analysis workflow. Regarding assembly, first, more than 200 cp-genomes were downloaded and blasted against the cp-genome of *S. miltiorrhiza.* Similar sequences in the cp-genome of *S. miltiorrhiza* were isolated and used as the basis of genome assembly. Second, the cp-genome of *Sesamum indicum* was selected for guiding the order of contigs because of its highest similarity with the *S. miltiorrhiza* cp-genome. Third, to fill the gaps in the assembly, isolated sequences and contigs were used to repeatedly search against SMRT reads of *S. miltiorrhiza* gDNA. Then, an initial assembly was obtained by extending the contigs, adding new reads, and conducting reassembly. Finally, the regions of junction between IRs and LSC (or SSC) were amplified and sequenced by Sanger sequencing and the final assembly was obtained by integrating the Sanger sequences into the initial assembly using Seqman (DNASTAR, WI). Strand-specific RNA sequencing was also conducted to determine the expression level of genes in the cp-genome of *S. miltiorrhiza.*

Further data analysis: The RNA-seq reads were mapped to the final assembly of *S. miltiorrhiza* cp-genome using Tophat to identify polycistrons and non-coding RNA (ncRNA) and to determine the content of protein-coding transcripts (cRNA) and ncRNA. Strand-specific real-time quantitative PCR (ss-qPCR) was also conducted to validate the results of RNA-seq. DNA modifications were predicted using the SMRT Portal software (v1.3.2). As a result, the authors identified 19 polycistronic transcripts containing 71 genes, which consisted of 58 protein-coding genes, four rRNA, and nine tRNA. Furthermore, 136 ncRNA transcripts were identified and classified into two categories, intergenic ncRNA and antisense ncRNA (asRNA). Using SMRT Portal 1.3.2, two DNA modification motifs and 2687 DNA modification sites were predicted. Interactions between asRNA and cRNA, DNA modification and gene expression were also analyzed. The results showed that the expression level of protein-coding genes was positively associated with that of asRNA, and the DNA modification was correlated with higher expression of ncRNA.

In addition to the software mentioned above, many kinds of newly developed software, such as GetOrganelle (Jin et al., 2020), Fast-Plast (https://github.com/mrmckain/Fast-Plast), CPGAVAS2 (Shi et al., 2019), and CPGview (Liu et al., 2023), are also available for *de novo* assembly, annotation, analysis and visualization of chloroplast genomes.

The mitochondrial genomes of medicinal plants are more complex than cp-genomes, which usually contain multiple conformations (isoforms) instead of circular molecules (Kozik et al., 2019; Wang et al., 2021c). Previously, many mitochondrial genome assemblies failed to obtain all the isoforms of the mitochondrial genome of medicinal plants because of the limitations of the methods (Kozik et al., 2019). With the help of TGS techniques, researchers successfully captured various conformations of the mitochondrial genomes of *Coriandrum sativum* (Wang et al., 2021c), *Scutellaria tsinyunensis* (Li et al., 2021b), and several other valuable medicinal plants, providing more complete and precise references for the further utilization of mitochondrial genome sequences.

### 3.2 Revelation of transcriptomes

Although combining whole genome sequencing with transcriptomic analysis is a useful strategy for characterizing the genetic information of medicinal plants, it is too expensive to obtain enough TGS and short reads data for *de novo* genome assembly. Species with high-quality reference genomes only account for a small proportion of medicinal plants. Therefore, finding an ideal strategy for characterizing genetic information of medicinal plants without reference genomes becomes important and promising. The emergence of TGS allowed researchers to obtain full-length transcriptomes at an isoform level at a low cost. Due to the application of TGS, mainly ONT and SMRT, to RNA sequencing, the transcriptome analysis methods have gradually been revolutionized (Zhao et al., 2019a). To date, more than 25 transcriptomes of medicinal plants, such as *S. miltiorrhiza* (Xu et al., 2015; Xu et al., 2016b), *Dendrobium officinale* (He et al., 2017), *Drynaria roosii* (Sun et al., 2018b), *Astragalus membranaceus* (Li et al., 2017a), and many other species have been revealed using TGS. Herein, we choose *A. membranaceus* as an example to demonstrate the specific process of applying TGS to transcriptomic analysis of species without reference genomes (Li et al., 2017a).

Sample and library preparation: In this study, taproots and leaves from *A. membranaceus* were collected, washed, and then stored in liquid nitrogen as samples, followed by RNA extraction using Spectrum Plant Total RNA Kit. The extracted RNAs were assessed using an Agilent 2100 Bioanalyzer, among which high-quality RNAs were utilized to prepare first-strand cDNA. Then, the first strand of cDNA was used to synthesize and amplify the second strand of cDNA. Finally, an Iso-seq library preparation was finished with 400 μl of cDNA from each sample.

Sequencing and data processing: The Iso-seq libraries were sequenced on the PacBio RSII with three SMRT cells for 1–2 kb libraries and five SMRT cells for 2–3 kb libraries. Assembly was not needed in this experiment. The raw data were processed with the standard RS_Iso-Seq protocol (SMRT Analysis 2.3). Specifically, according to the results of polyA tails and primers detection, 494,408 reads of inserts (ROIs) for leaf tissue and 500,007 ROIs for root tissue in the raw data were classified as full-length and non-full-length reads. The authors obtained 115,725 full-length consensus sequences for leaf tissue and 102,334 for root tissue from full-length ROIs and clustered them into different isoforms, followed by polishing with non-full-length ROIs. Full-length consensus sequences with more than 99% accuracy were classified as high-quality (HQ) transcripts, while other sequences were classified as low-quality (LQ) transcripts using Quiver. As a result, researchers generated 75,816 HQ transcripts and 39,909 LQ transcripts for leaf tissue and 73,755 HQ transcripts and 28579 LQ transcripts for root tissue. Finally, HQ and LQ transcripts were corrected with an Illumina RNA-seq paired-end data set followed by redundancy removal using the CD-HITv4.6 package.

Further data analysis: For isoform identification, the non-redundant transcripts were clustered into families using the Coding GENome reconstruction Tool (Cogent v1.4). Finally, these transcript families were reconstructed as one or more unique transcript models through the De Bruijn graph method. Mapping the non-redundant transcripts to the unique transcript models, splicing junctions for transcripts were examined. Transcription isoforms of unique transcript models were identified by collapsing transcripts with identical splicing junctions, and SUPPA was used to detect alternative splicing events.

Functional annotation: Four protein databases (UniProtKB_Viridiplan-tae, UniProtKB_MEDTR, UniProtKB_SOYBN, and the curated soybean reference protein annotation) were used for functional annotation of unique transcript models using BlASTX (NCBI-BLAST v2.2.27+) and unique transcript models were then classified using GO and KEGG based on the best hit from UniProtKB_SOYBN.

Long non-coding RNA (LncRNA) identification: After removing annotated transcripts and filtering out unique transcript models with ORFs with a length of more than 100 amino acids or 50 amino acids at the end(s) internally, LncRNAs were annotated using Coding Potential Calculator v0.9r2 to assess ORF-filtered unique transcripts models.

Multiple cutting-edge transcriptomic-analysis software have recently been developed, such as 3GOLD (Logan et al., 2022) and MeShClust v3.0 (Girgis, 2022) for high-speed or high-quality sequence clustering, RATTLE (de la Rubia et al., 2022) for reference-free reconstruction and quantification of transcripts and NanoSplicer (You et al., 2022) for identifying splice junctions.

The full-length transcriptomes obtained using TGS also provide valuable resources about the expression pattern and isoforms of many functional genes associated with the biosynthesis of active components in medicinal plants.

Alternative splicing events of muti-exon genes in multicellular eukaryotes can enhance the functional diversity of the encoded proteins and regulate gene expression through complex post-transcriptional mechanisms (Reddy et al., 2013). TGS, with its long read lengths, can deliver high yields of long, full-length RNA or cDNA, supporting the quantification of genes and complete transcriptome analysis at the isoform level, which is especially useful for species without a reference genome (Xu et al., 2015).

### 3.3 Molecular identification

Current methods were insufficient for the quality control of multiple herbal ingredients in traditional Chinese patent medicines. Combining TGS with DNA barcoding has made it possible to monitor the quality of traditional Chinese patent medicines effectively and affordably, as verified in the study of Yimu Wan (Jia et al., 2017) and Jiuwei Qianghuo Wan (Xin et al., 2018).

In this section, we describe the molecular identification of traditional Chinese patent medicine ‘Yimu Wan’ (YMW) as an example (Jia et al., 2017). In the selected study, two reference samples of YMW, RF01 and RF02, were used to establish a standard method for identification, which was then successfully applied to commercial YMW samples.

Sample preparation: The reference samples RF01 and RF02 were made in the laboratory under the guidance of the Chinese Pharmacopoeia. RF02 was formulated by weighing 10 g of the mixed powder of *Leonurus japonicas*, *Angelica sinensis*, *Ligusticum chuanxiong*, *Aucklandia lappa* and other recorded proportions. *Panax ginseng* powder was then added to one RF02 sample as a biological indicator. For RF01, only *P. ginseng* was spared. Finally, pills were molded by mixing these two samples with double-distilled water. One-hundred-twenty milligrams of the sample RF02 was used to isolate gDNA, the quality of obtained gDNA was assessed by Nanodrop 2000, and the DNA concentrations were determined using an Agilent 2100 bioanalyzer.

Library preparation: Before library construction, the gDNA underwent PCR and purification. For the PCR process, different primers were added to distinct samples to amplify ITS2 and *psbA-trnH*. And the universal ITS2 and *psbA-trnH* primers were ligated with two tags (5 bp) to differentiate the sequences from different regions. The PCR process was conducted as described in the Chinese Pharmacopoeia. After purification, the PCR products were used to construct a SMRT sequencing library using the SMRTbell Template Prep Kit 1.0.

Sequencing and data processing: ITS2 and *psbA-trnH* amplicon sequencing were conducted on the PacBio SMRT instrument. CCS sub-read datasets were obtained using SMRT Analysis Server 2.3.0 provided by PacBio. The CCS reads from RF02 were extracted according to the tags mentioned above, which were used to construct data libraries using Perl scripts. The CCS reads were clustered followed by removing redundant sequences, and then identified in the DNA Barcoding System for Identifying Herbal Medicine using BLAST.

Validating the standard method: To validate its replicability, the same procedure as the quality control protocol established above was conducted with RF01.

Applying the standard method to commercial YMW: Three batches of YMW produced by the same manufacturer were randomly bought from various drug stores. The same sample preparation and testing methods as RF01 and RF02 were used for these samples. As a result, this research successfully developed an effective protocol to assess the quality of traditional Chinese patent medicines using PacBio SMRT sequencing.

## 4 Advantages and challenges

### 4.1 Advantages

According to previous studies (**Supplementary Table 1**), nuclear genomes of medicinal plants are usually diploids or polyploids with large genome sizes, high heterozygosity, and high repeat sequences proportion. It is also demonstrated that the GC content of nuclear genomes of many medicinal plants is generally lower than 50% on average and unevenly distributed in different chromosomes (Shang et al., 2020; Sun et al., 2020; Wu et al., 2021b; Li et al., 2022a; Xu et al., 2022a). These features have brought traditional short reads methods under challenge. Specifically, large repetitive and high/low GC content regions principally account for the misassemblies and gaps in the final NGS genome assemblies (Salzberg and Yorke, 2005; Schmidt and Pearson, 2016; Guo et al., 2018). As for genomic variations, although precise detection of single nucleotide polymorphisms (SNPs) and indels can be achieved by NGS, structure variations (SVs) remain difficult to detect. Moreover, because the distance between variations exceeds the length of short reads, it is difficult for NGS techniques to link individual SNPs and indels together and phase haplotypes and alleles (van Dijk et al., 2018). However, these obstacles can be overcome by TGS. With long read lengths, TGS can span most of the repeat regions and large SVs in medicinal plant genomes. Genomic variations, including SNPs, indels, and SVs, are also naturally connected in the same long read, making it much easier to phase alleles or haplotypes (Stander et al., 2021). Several polypoid medicinal plants [such as *Triadica sebifera* (4n=88) (Luo et al., 2022), *Rehmannia glutinosa* (4n=56) (Ma et al., 2021), *Aquilegia oxysepala* var. *kansuensis* (4n=28) (Xie et al., 2020)], species with high genome heterozygosity [such as *Aloe vera* (11.3%) (Jaiswal et al., 2021), *Curcuma longa* (4.83%) (Chakraborty et al., 2021), *Gar. jasminoides* (2.2%) (Xu et al., 2020b)], and species with an extremely high proportion of repeat sequences [such as *Allium sativum* (91.3%) (Sun et al., 2020), *Panax notoginseng* (88.2%) (Yang et al., 2021b)], were all sequenced and assembled using SMRT, ONT, or both, yielding many high-quality chromosome-level assemblies.

Regarding transcriptome research, first, the vast majority of eukaryotic genes do not strictly conform to the ‘one gene-one transcript’ pattern. Instead, they often have several different isoforms. The application of TGS allows researchers to obtain full-length transcripts at an isoform level, even if a reference genome is not available (Li et al., 2017a). Second, with low GC bias, SMRT and ONT also allow more precise quantification of the expression level of genes than NGS techniques, facilitating research into expression patterns of important genes.

### 4.2 Challenges

First, when attempting nuclear genome sequencing of medicinal plants, it is difficult for ONT to achieve both high accuracy and extremely long read lengths when used alone. For example, when applied to the sequencing of the polyploid genome of *Veratrum dahuricum*, ONT produced ultra-long reads. However, by mapping the NGS reads against the ONT assemblies and a SMRT CCS assembly, researchers found that the coverage of three ONT assemblies ranged from 49.15% to 76.31%, much smaller than that of the SMRT CCS assembly (99.53%) (Zeng et al., 2022). A hybrid sequencing approach seems to be a good resolution because it has been shown in many medicinal organisms (Jain et al., 2018; Song et al., 2018; Wang et al., 2018) that combining NGS short reads with ONT can improve both the accuracy and completeness of obtained assemblies. However, this strategy also greatly increases the sequencing costs.

Second, when applied to chloroplast genome sequencing of medicinal plants, two most used sample preparation methods are isolating chloroplasts from the plant tissue (Li et al., 2014; Wu et al., 2014) and extracting chloroplast sequences from sequencing data of total DNA (Chen et al., 2014). However, the former method is difficult for most researchers who are not specialists in chloroplast extraction, while the latter method is expensive because it needs to sequence the whole genome of medicinal plants. For this reason, we consider that TGS is a poor choice for sequencing chloroplast genomes of medicinal plants.

## 5 Discussion

As this review has demonstrated, applying TGS, mainly SMRT and ONT, can greatly promote the development of herbal genomics. So far, the nuclear genomes of more than 100 medicinal plants have been sequenced using TGS, a large proportion of which were assembled to a chromosome level, while the organelle genomes of some important medicinal organisms have also been precisely assembled using TGS data. In addition, TGS is revolutionizing how transcriptomes of medicinal plants are analyzed by enabling the acquisition of full-length transcriptomes at an isoform level without a reference genome. Furthermore, TGS combined with DNA barcoding is also an effective and affordable approach to monitoring the compositions of traditional Chinese patent medicines. In a word, TGS has greatly contributed to herbal genomics and enriched the genetic information of organism-derived species. However, studies of molecular identification using TGS are still rare, making it a promising field to study further. Apart from the fields mentioned above, the epigenomics of medicinal organisms is also promising because TGS can directly detect methylations of DNA and RNA molecules. In addition, assembling gapless T2T genomes using PacBio HiFi reads and ONT ultra-long reads is a new trend in the genomic research of animals and parasites, and it greatly increased our understanding of telomere and centromere regions. T2T genomes are still rarely reported for medicinal organisms, which should be a focus of future work.

## Author contributions

LG and JS designed the review. LG wrote the manuscript. WX, TX, and JS revised and edited the manuscript. All authors contributed to the article and approved the submitted version.

## Footnotes

1
https://www.pacb.com/wp-content/uploads/Procedure-checklist-Preparing-whole-genome-and-metagenome-libraries-using-SMRTbell-prep-kit-3.0.pdf

2
https://www.pacb.com/technology/hifi-sequencing/

3
https://www.pacb.com/wp-content/uploads/Overview-Sequel-Systems-Application-Options-and-Sequencing-Recommendations.pdf

4
https://nanoporetech.com/products/kits

5
https://community.nanoporetech.com/protocols/genomic-dna-by-ligation-sqk-lsk114

6
https://nanoporetech.com/products/voltrax

7
https://community.nanoporetech.com/docs/sequence/sequencing_software/data-analysis

8
https://nanoporetech.com/products

9
https://nanoporetech.com/products/flongle

10 1^0^ https://www.pacb.com/technology/hifi-sequencing/sequel-system,

11 1^1^ https://www.pacb.com/revio/

12 1^2^ https://nanoporetech.com/products

13 1^3^ https://nanoporetech.com/products/kits

14 1^4^ https://www.illumina.com.cn/systems/sequencing-platforms.html
