# Supplementary material for: Application of third-generation sequencing to herbal genomics
Source: Front Plant Sci. 2023 Mar 7;14:1124536. doi: 10.3389/fpls.2023.1124536 (PMC10027759; doi:10.3389/fpls.2023.1124536)
Supplement: Supplementary file 1 [file Table_1.docx]

**Supplementary Table 1 Nuclear genome assemblies obtained using TGS techniques**

| **Family** | **Species** | **Tech-nique** | **Total size /Mb** | **Contig N50/kb** | **Scaffold N50/Mb** | **Repeat sequence** | **Protein-coding genes** | **Reference** |
| --- | --- | --- | --- | --- | --- | --- | --- | --- |
| **Dicotyledoneae** |  |  |  |  |  |  |  |  |
| Lamiaceae | *Salvia miltiorrhiza* | P,I | 641 | 82.8 | 1.2 | 53.58% | 34,598 | ([Zhang et al., 2015](#_ENREF_111)) |
|  | *Salvia miltiorrhiza* | P,I,R | 538 | 12.38 | 0.051 | 54.44% | 30,478 | ([Xu et al., 2016](#_ENREF_93)) |
|  | *Salvia miltiorrhiza* | P,I,H | 595 | 2,705 | 69.8 | 64.84% | 32,483 | ([Song et al., 2020](#_ENREF_66)) |
|  | *Salvia officinalis* | P,I,H | 480 | 3,050 | 63.8 | 61.67% | 31,713 | ([Li et al., 2022a](#_ENREF_35)) |
|  | *Salvia bowleyana* | P,I,H | 462 | 1,180 | 57.96 | 58.70% | 44,044 | ([Zheng et al., 2021](#_ENREF_118)) |
|  | *Mentha longifolia* | P,I,G | 353 | 4.47 | **/** | **/** | 35,597 | ([Vining et al., 2017](#_ENREF_78)) |
|  | *Scutellaria baicalensis* | P,I, 10×,H | 387 | 880.6 | 33.2 | 55.15% | 28,524 | ([Zhao et al., 2019](#_ENREF_117)) |
|  | *Scutellaria baicalensis* | O,I,H | 377 | 2,100 | 40.8 | **/** | 33,414 | ([Xu et al., 2020a](#_ENREF_98)) |
|  | *Scutellaria baicalensis* | P,I,H | 377 | 1,800 | 40.57 | 57.73% | 31,896 | ([Hu et al., 2022](#_ENREF_23)) |
|  | *Scutellaria barbata* | P,I,H | 353 | 2,500 | 23.7 | **/** | 41,697 | ([Xu et al., 2020a](#_ENREF_98)) |
|  | *Perilla frutescens* | P,H,D | 1,259 | 41,560 | 63.33 | 68.84% | 76,825 | ([Tamura et al., 2023](#_ENREF_72)) |
|  | *Pogostemon cablin* | O,P,I,H | 1,940 | 7,970 | **/** | 65.94% | 109,696 | ([Shen et al., 2022b](#_ENREF_63)) |
| Compositae | *Arctium lappa* | P,I,H | 1,790 | 6,883 | 91.64 | 68.46% | 32,771 | ([Yang et al., 2022b](#_ENREF_105)) |
|  | *Stevia rebaudiana* | O,I,H | 1,416 | 616.9 | 106.6 | 80.11% | 44,143 | ([Xu et al., 2021](#_ENREF_96)) |
|  | *Chrysanthemum nankingense* | O,I | 2,530 | 130.7 | **/** | 69.6% | 56,870 | ([Song et al., 2018](#_ENREF_64)) |
|  | *Chrysanthemum makinoi* | P,O,I,H | 3,100 | 258.2 | 330.0 | 80.04% | 95,074 | ([van Lieshout et al., 2022](#_ENREF_76)) |
|  | *Carthamus tinctorius* | P,I,H,G | 1,060 | 21,230 | 88.21 | 60.13% | 33,343 | ([Wu et al., 2021](#_ENREF_88)) |
|  | *Artemisia annua* | P,I,R | 1,740 | 18.95 | 0.1049 | 61.57% | 63,226 | ([Shen et al., 2018](#_ENREF_62)) |
|  | *Artemisia annua* strain LQ-9 haplotype 0 | P,I,H,Bio | 1,549 | **/** | 70.99 | 74.94% | 54,347 | ([Liao et al., 2022](#_ENREF_41)) |
|  | *Artemisia annua* strain LQ-9 haplotype 1 | P,I,H,Bio | 1,474 | **/** | 39.03 | 73.51% | 44,457 | ([Liao et al., 2022](#_ENREF_41)) |
|  | *Artemisia annua* strain HAN1 haplotype 0 | P,I,H,Bio | 1,590 | **/** | 44.16 | 67.10% | 45,285 | ([Liao et al., 2022](#_ENREF_41)) |
|  | *Artemisia annua* strain HAN1 haplotype 1 | P,I,H,Bio | 1,383 | **/** | 20.61 | 68.23% | 51,821 | ([Liao et al., 2022](#_ENREF_41)) |
|  | *Artemisia argyi* | P,I,H,M | 8,030 | 6.25 | 206.4 | 73.59% | 279,294 | ([Miao et al., 2022](#_ENREF_50)) |
|  | *Artemisia argyi* | P,I,H | 3,890 | 1,450 | 214 | 81.03% | 62,844 | ([Chen et al., 2023](#_ENREF_5)) |
| Leguminosae | *Vigna umbellata* | P,I | 415 | **/** | 0.0781 | **/** | 31,276 | ([Kaul et al., 2022](#_ENREF_34)) |
|  | *Dalbergia odorifera* | P,I,10× | 638 | 5,920 | 56.16 | 54.17% | 30,310 | ([Hong et al., 2020](#_ENREF_22)) |
|  | *Glycyrrhiza uralensis* | P,I | 379 | 7.324 | 0.1093 | 36.48% | 34,445 | ([Mochida et al., 2017](#_ENREF_51)) |
|  | *Glycyrrhiza uralensis* | P,I,H | 449 | 36,020 | 60.2 | 61.7% | 32,941 | ([Rai et al., 2022](#_ENREF_58)) |
|  | *Spatholobus suberectus* | P,I,10×,H | 798 | 2,100 | 86.99 | 47.82% | 31,634 | ([Qin et al., 2019](#_ENREF_56)) |
|  | *Abrus cantoniensis* | O,H | 381 | 18,930 | 18.95 | 45.12% | 25,058 | ([Xu et al., 2022b](#_ENREF_95)) |
|  | *Senna tora* | P,I,H | 526 | 4,030 | 41.7 | 53.9% | 45,268 | ([Kang et al., 2020c](#_ENREF_33)) |
|  | *Astragalus membranaceus* var. *mongholicus* | P,I,H | 1,470 | 9,790 | 181.02 | 70.72% | 27,868 | ([Chen et al., 2022](#_ENREF_6)) |
|  | *Entada phaseoloides* | O,I,H,M | 456 | 6,343 | 30.89 | 52.7% | 24,894 | ([Lin et al., 2022](#_ENREF_42)) |
| Cucurbitaceae | *Luffa acutangula* | P,I,H | 735 | **/** | 0.7861 | 62.17% | 32,233 | ([Pootakham et al., 2021](#_ENREF_54)) |
|  | *Luffa cylindrica* | P,I,H | 690 | **/** | 0.5786 | 56.78% | 43,828 | ([Pootakham et al., 2021](#_ENREF_54)) |
|  | *Luffa cylindrica* | P,I,H | 669 | 4,816 | 48.66 | 62.18% | 31,661 | ([Zhang et al., 2020](#_ENREF_114)) |
|  | *Luffa cylindrica* | P,I,10×,H | 656 | 8,800 | 48.76 | 63.81% | 25,508 | ([Wu et al., 2020](#_ENREF_87)) |
|  | *Gynostemma pentaphyllum* | O,H,D | 609 | 5,050 | 56.38 | 60.23% | 26,588 | ([Zhang et al., 2023](#_ENREF_116)) |
|  | *Herpetospermum pedunculosum* | P,I,H | 804 | 24,390 | 71.40 | 72.08% | 23,924 | ([Yang et al., 2023](#_ENREF_106)) |
|  | *Momordica charantia* var. *abbreviata* | P,I,H | 296 | 26,380 | 25.38 | 45.59% | 19,895 | ([Fu et al., 2023](#_ENREF_15)) |
| Umbelliferae | *Angelica sinensis* | P,H | 2,370 | 25,420 | 193.9 | 77.74% | 43,202 | ([Han et al., 2022](#_ENREF_21)) |
|  | *Bupleurum chinense* haplotype 0 | P,I,H | 621 | 16,860 | 92.25 | 53.94% | 45,909 | ([Zhang et al., 2022a](#_ENREF_113)) |
|  | *Bupleurum chinense* haplotype 1 | P,I,H | 600 | 23,900 | 102.7 | 53.47% | 35,805 | ([Zhang et al., 2022a](#_ENREF_113)) |
|  | *Coriandrum sativum* | P,I,H | 2,119 | 604.1 | 161.0 | 80.58% | 40,747 | ([Song et al., 2021](#_ENREF_65)) |
| Rosaceae | *Rubus idaeus* | P,I,H | 292 | 241.8 | 34.49 | **/** | 39,448 | ([Davik et al., 2022](#_ENREF_10)) |
|  | *Rubus occidentalis* | P,I,H | 290 | 5,100 | 41.1 | **/** | 34,545 | ([VanBuren et al., 2018](#_ENREF_77)) |
|  | *Eriobotrya japonica* | O,I,H | 760 | 5,020 | 39.7 | 59.17% | 45,743 | ([Jiang et al., 2020](#_ENREF_28)) |
|  | *Eriobotrya japonica* | P,I,H | 761 | 3,980 | 43.16 | 85.90% | 43,996 | ([Su et al., 2021](#_ENREF_67)) |
| Araliaceae | *Eleutherococcus senticosus* | P,I,H | 1,300 | 309.4 | 50.79 | 73.55% | 36,372 | ([Yang et al., 2021b](#_ENREF_107)) |
|  | *Panax notoginseng* | P,O,H | 2,254 | 220.9 | **/** | 79.07% | 39,452 | ([Fan et al., 2020](#_ENREF_13)) |
|  | *Panax notoginseng* | P,I,H | 2,660 | 1,120 | 216.5 | 85.85% | 37,606 | ([Jiang et al., 2021](#_ENREF_29)) |
|  | *Panax notoginseng* | P,I,H | 2,410 | 1,450 | 196.3 | 88.20% | 47,870 | ([Yang et al., 2021b](#_ENREF_107)) |
| Acanthaceae | *Strobilanthes cusia* | P,I,H | 914 | 35,590 | 68.44 | 73.94% | 32,974 | ([Hu et al., 2021](#_ENREF_24)) |
|  | *Andrographis paniculate* | P,I,H | 284 | 5,150 | **/** | 57.35% | 24,015 | ([Liang et al., 2020](#_ENREF_40)) |
|  | *Andrographis paniculate* | P,I | 269 | 388.9 | **/** | 53.3% | 25,428 | ([Sun et al., 2019](#_ENREF_70)) |
| Rubiaceae | *Morinda officinalis* | O,I,,H,M | 485 | 4,214 | 40.97 | 58.04% | 27,698 | ([Wang et al., 2021a](#_ENREF_81)) |
|  | *Ophiorrhiza pumila* | P,I,H,Bio | 440 | 18,490 | 40.06 | 58.17% | 32,389 | ([Rai et al., 2021](#_ENREF_57)) |
|  | *Gardenia jasminoides* | O,I,H | 535 | 1,000 | 44 | 62.2% | 35,967 | ([Xu et al., 2020b](#_ENREF_100)) |
| Lythraceae | *Punica granatum* | P,I,10×,Bio | 346 | 6,800 | 16.12 | 51.7% | 29,435 | ([Roopa Sowjanya et al., 2022](#_ENREF_60)) |
|  | *Punica granatum* | O,I | 362 | 61.03 | 40.75 | 26.68% | 30,803 | ([Usha et al., 2022](#_ENREF_74)) |
|  | *Punica granatum* | P,I,H | 320 | 4,490 | 39.96 | 50.93% | 33,594 | ([Luo et al., 2020](#_ENREF_45)) |
| Rutaceae | *Poncirus trifoliata* | P,I,H | 265 | 842.8 | 27.7 | **/** | 25,538 | ([Peng et al., 2020](#_ENREF_53)) |
|  | *Citrus grandis* | O,B | 359 | **/** | 9.12 | **/** | 30,238 | ([Xian et al., 2022](#_ENREF_91)) |
|  | *Citrus grandis* | P,I | 345 | 2,183 | 4.21 | **/** | 30,123 | ([Wang et al., 2017](#_ENREF_83)) |
| Theaceae | *Camellia sinensis* | P,I,S | 3,100 | 67.07 | 1.39 | 64% | 33,932 | ([Wei et al., 2018](#_ENREF_86)) |
|  | *Camellia sinensis* | O,I,H | 3,080 | 723.7 | 207.7 | 86.77% | 34,896 | ([Wang et al., 2022a](#_ENREF_79)) |
| Caprifoliaceae | *Lonicera japonica* | P,I,H | 886 | 1,579 | 79.57 | 64.76% | 39,320 | ([Yu et al., 2022](#_ENREF_110)) |
|  | *Lonicera japonica* | O,I,H | 843 | 2,100 | 84.4 | 58.21% | 35,967 | ([Pu et al., 2020](#_ENREF_55)) |
| Ranunculaceae | *Coptis chinensis* | P,I,H | 958 | 1,580 | 4.53 | 62.23% | 34,109 | ([Chen et al., 2021](#_ENREF_4)) |
|  | *Coptis chinensis* | O,I,H | 937 | 806.6 | **/** | 62.5% | 41,004 | ([Liu et al., 2021](#_ENREF_44)) |
| Eucommiaceae | *Eucommia ulmoides* | P,I,B | 1,180 | 17.06 | 1.88 | 61.24% | 26,723 | ([Wuyun et al., 2018](#_ENREF_89)) |
|  | *Eucommia ulmoides* | P,I,H | 948 | 13,160 | 53.15 | 62.50% | 26,001 | ([Li et al., 2020b](#_ENREF_39)) |
| Cucurbitaceae | *Siraitia grosvenorii* | P,I | 470 | 432.4 | **/** | 50.54% | 30,565 | ([Xia et al., 2018](#_ENREF_90)) |
|  | *Momordica charantia* | P,I,G | 303 | 9,900 | 25.37 | 52.52% | **/** | ([Matsumura et al., 2020](#_ENREF_49)) |
| Lardizabalaceae | *Akebia trifoliata* | P,I,H | 653 | 1,570 | 35.75 | 70.1% | 24,138 | ([Zhong et al., 2022](#_ENREF_119)) |
|  | *Akebia trifoliata* subsp. *australis* | P,I,H | 682 | 6,200 | 43.11 | 71.18% | 25,598 | ([Huang et al., 2021](#_ENREF_25)) |
| Papaveraceae | *Papaver somniferum* | P,O,I,10×,G | 2,720 | 1,770 | 204 | 70.9% | 51,213 | ([Guo et al., 2018](#_ENREF_19)) |
|  | *Corydalis tomentella* | P,I,H | 249 | 2,520 | **/** | **/** | 37,808 | ([Xu et al., 2022d](#_ENREF_99)) |
| Euphorbiaceae | *Vernicia fordii* | P,I,H | 1,120 | **/** | 87.15 | 73.34% | 28,422 | ([Zhang et al., 2019](#_ENREF_112)) |
|  | *Phyllanthus cochinchinensis* | O,I,H | 285 | 10,320 | **/** | 59.12% | 20,836 | ([Zhang et al., 2022b](#_ENREF_115)) |
| Gentianaceae | *Gentiana macrophylla* | O,I,H | 1,790 | 720.8 | 122.7 | 73.47% | 55,337 | ([Zhou et al., 2022a](#_ENREF_120)) |
|  | *Gentiana dahurica* | O,I,H | 1,417 | 1,300 | 113.3 | 70.25% | 37,988 | ([Li et al., 2022b](#_ENREF_38)) |
| Oleaceae | *Forsythia suspensa* | O,I | 737 | **/** | 7.3 | 54.5% | 33,062 | ([Li et al., 2020a](#_ENREF_37)) |
| Guttiferae | *Hypericum perforatum* | P,I,10× | 374 | 1,410 | 2.31 | 46.9% | 29,150 | ([Zhou et al., 2021](#_ENREF_121)) |
| Magnoliaceae | *Magnolia biondii* | P,10×,H | 2,232 | 269.1 | 92.86 | 66.48% | 47,547 | ([Dong et al., 2021](#_ENREF_12)) |
| Paeoniaceae | *Paeonia suffruticosa* | P,B | 13,790 | 49.9 | **/** | 80.24% | 34,854 | ([Lv et al., 2020](#_ENREF_46)) |
| Celastraceae | *Tripterygium wilfordii* | P,I,10×,H | 348 | 4,360 | 13.52 | 52.36% | 28,321 | ([Tu et al., 2020](#_ENREF_73)) |
| Myrtaceae | *Psidium guajava* | P,I,H | 444 | 15.8 | 40.4 | 43.55% | 25,601 | ([Feng et al., 2021](#_ENREF_14)) |
| Loranthaceae | *Taxillus chinensis* | O,I,H | 522 | 3,800 | 56.90 | 55.8% | 33,894 | ([Fu et al., 2022](#_ENREF_16)) |
| Euphorbiaceae | *Euphorbia lathyris* | O,I,H | 989 | 32,590 | 95.72 | 65.90% | 36,342 | ([Wang et al., 2021b](#_ENREF_82)) |
| Orobanchaceae | *Rehmannia glutinosa* | O,I,H | 2,490 | 658.9 | 70.28 | >67% | 48,475 | ([Ma et al., 2021](#_ENREF_47)) |
| Boraginaceae | *Lithospermum erythrorhizon* | O,I | 367 | 314.3 | **/** | 51.78% | 27,720 | ([Auber et al., 2020](#_ENREF_1)) |
| Sapindaceae | *Acer truncatum* | P,I,10×,H | 633 | 773.2 | 46.36 | 61.75% | 28,438 | ([Ma et al., 2020](#_ENREF_48)) |
| Convolvulaceae | *Cuscuta australis* | P,I | 265 | 3,630 | 5.95 | 58.5% | 19,671 | ([Sun et al., 2018](#_ENREF_69)) |
| Solanaceae | *Datura stramonium* | O,I | 2,100 | 13.1 | 0.1641 | 61% | 52,149 | ([Rajewski et al., 2021](#_ENREF_59)) |
| Thymelaeaceae | *Aquilaria sinensis* | O,I,H | 727 | 1,100 | 88.78 | 59.13% | 29,203 | ([Ding et al., 2020](#_ENREF_11)) |
| Cannabaceae | *Cannabis sativa* | P,I,H | 808 | 513.6 | 83.00 | 74.75% | 38,828 | ([Gao et al., 2020](#_ENREF_17)) |
| Cruciferae | *Isatis indigotica* | P,I,H | 294 | 1,180 | 36.16 | 53.27% | 30,323 | ([Kang et al., 2020a](#_ENREF_31)) |
| Hippocastanaceae | *Aesculus wilsonii* | O,I,H | 579 | 3,750 | 28.02 | 54.46% | 46,914 | ([Ye et al., 2022](#_ENREF_108)) |
| Aristolochiaceae | *Aristolochia contorta* | O,I,H | 209 | 2,630 | 30.38 | 38.26% | 18,311 | ([Cui et al., 2022](#_ENREF_9)) |
| Nyssaceae | *Camptotheca acuminata* | P,I,H | 415 | 1,474 | 18.28 | 37.55% | 27,940 | ([Kang et al., 2021](#_ENREF_30)) |
| Lauraceae | *Cinnamomum camphora* | P,I,H | 704 | 2,190 | 3.17 | 59.64% | 36,411 | ([Wang et al., 2022c](#_ENREF_84)) |
| Berberidaceae | *Epimedium pubescens* | O,I,H | 3,340 | 871.8 | **/** | 66.93% | 44,722 | ([Shen et al., 2022a](#_ENREF_61)) |
| Asclepiadaceae | *Marsdenia tenacissima* | P,I,H | 382 | 6,570 | **/** | 58.3% | 21,899 | ([Zhou et al., 2022b](#_ENREF_122)) |
| Apocynaceae | *Voacanga thouarsii* | O,I | 1,354 | 2,910 | 3.04 | **/** | 33,300 | ([Cuello et al., 2022](#_ENREF_8)) |
| **Monocot-yledoneae** |  |  |  |  |  |  |  |  |
| Zingiberaceae | *Zingiber officinale* haplotype 1 | P,I,H | 1,530 | 4,680 | 141.5 | 56.90% | 39,217 | ([Li et al., 2021](#_ENREF_36)) |
|  | *Zingiber officinale* haplotype 0 | P,I,H | 1,510 | 5,280 | 143.0 | 56.70% | 38,090 | ([Li et al., 2021](#_ENREF_36)) |
|  | *Amomum tsaoko* | P,I | 2,700 | 2,450 | **/** | 89.15% | 54,379 | ([Sun et al., 2022](#_ENREF_68)) |
|  | *Curcuma longa* | O,I,10× | 1,020 | 100.6 | **/** | 64.16% | 50,401 | ([Chakraborty et al., 2021](#_ENREF_3)) |
|  | *Curcuma longa* | P,I,H | 1,110 | 2,340 | 50.12 | 69.99% | 49,612 | ([Yin et al., 2022](#_ENREF_109)) |
|  | *Wurfbainia villosa* | O,I,H | 2,800 | 9,130 | 109.9 | 87.23% | 42,588 | ([Yang et al., 2022a](#_ENREF_103)) |
|  | *Boesenbergia rotunda* | P,I | 2,347 | 123.9 | 0.3947 | 72% | 71,072 | ([Taheri et al., 2022](#_ENREF_71)) |
| Orchidaceae | *Dendrobium Huoshanense* | P,I,H | 1,285 | 598 | 71.79 | 79.38% | 21,070 | ([Han et al., 2020](#_ENREF_20)) |
|  | *Dendrobium nobile* | P,H,M | 1,199 | 1,618 | 64.46 | 61.07% | 29,476 | ([Xu et al., 2022a](#_ENREF_94)) |
|  | *Dendrobium ofﬁcinale* | P,I | 1,350 | 25.1 | 0.0764 | 63.33% | 35,567 | ([Yan et al., 2015](#_ENREF_102)) |
|  | *Dendrobium ofﬁcinale* | P,I,H | 1,230 | 1,440 | 63.07 | 64.39% | 27,631 | ([Niu et al., 2021](#_ENREF_52)) |
|  | *Gastrodia elata* | P,I,H | 1,045 | 9.18 | 50.6 | 74.92% | 18,844 | ([Bae et al., 2022](#_ENREF_2)) |
|  | *Gastrodia elata* | P,I,M | 1,040 | 16,870 | **/** | 77.94% | 17,895 | ([Wang and Shahid, 2022](#_ENREF_85)) |
|  | *Bletilla striata* haplotype A | P,I,H | 2,369 | 1,650 | 146.39 | **/** | 26,673 | ([Jiang et al., 2022](#_ENREF_27)) |
|  | *Bletilla striata* haplotype B | P,I,H | 2,431 | 1,660 | 150.22 | **/** | 26,891 | ([Jiang et al., 2022](#_ENREF_27)) |
|  | *Cremastra appendiculata* | P,B,H | 2,360 | 677.9 | 80.16 | 59.15% | 20,991 | ([Wang et al., 2022b](#_ENREF_80)) |
| Gramineae | *Coix aquatica* | P,I,H | 1,619 | 751 | 2.24 | 75.39% | 39,629 | ([Guo et al., 2020](#_ENREF_18)) |
|  | *Coix lacryma-jobi* | P,I,Bio,H | 1,730 | 3,190 | 13.98 | 77.7% | 44,485 | ([Liu et al., 2020](#_ENREF_43)) |
|  | *Coix lacryma-jobi* | P,I | 1,280 | **/** | 0.5943 | 77.0% | 39,574 | ([Kang et al., 2020b](#_ENREF_32)) |
| Dioscoreaceae | *Trichopus zeylanicus* | P,I | 714 | 290 | 0.433 | 47.4% | 34,452 | ([Vadakkemukadiyil Chellappan et al., 2019](#_ENREF_75)) |
| Palmae | *Areca catechu* | P,I,H | 2,510 | 872 | 172.0 | **/** | 31,571 | ([Yang et al., 2021a](#_ENREF_104)) |
| Liliaceae | *Aloe vera* | O,I | 12,930 | **/** | 0.0146 | **/** | 86,177 | ([Jaiswal et al., 2021](#_ENREF_26)) |
| Agavaceae | *Dracaena cochinchinensis* | P,I,H,10× | 1,210 | 1,100 | 50.06 | **/** | 31,619 | ([Xu et al., 2022c](#_ENREF_97)) |
| **Gymno-spermae** |  |  |  |  |  |  |  |  |
| Taxaceae | *Taxus wallichiana* | O,I,H | 10,900 | 8,600 | 987 | 85% | 44,008 | ([Cheng et al., 2021](#_ENREF_7)) |
|  | *Taxus chinensis* var. *mairei* | P,I,H | 10,230 | 2,440 | **/** | 76.09% | 42,746 | ([Xiong et al., 2021](#_ENREF_92)) |
| **Lycophytina** |  |  |  |  |  |  |  |  |
| Selaginellaceae | *Selaginella tamariscina* | P,I | 301 | 201.2 | 0.4077 | 60.58% | 27,761 | ([Xu et al., 2018](#_ENREF_101)) |

P, PacBio SMRT; O, Oxford Nanopore; I, Illumina; S, Sanger;10×, 10×Genomics; R, Roche/454; M, MGISEQ-2000; B, BGISEQ-500; D, DNB-SEQ; H, Hi-C; G, Genetic linkage map; Bio, Bionano optical maps; /, not reported.

References

Auber, R.P., Suttiyut, T., McCoy, R.M., Ghaste, M., Crook, J.W., Pendleton, A.L., et al. (2020). Hybrid de novo genome assembly of red gromwell (*Lithospermum erythrorhizon*) reveals evolutionary insight into shikonin biosynthesis. *Hortic Res* 7(1)**,** 82. doi: 10.1038/s41438-020-0301-9.

Bae, E.K., An, C., Kang, M.J., Lee, S.A., Lee, S.J., Kim, K.T., et al. (2022). Chromosome-level genome assembly of the fully mycoheterotrophic orchid *Gastrodia elata*. *G3 (Bethesda)* 12(3)**,** jkab433. doi: 10.1093/g3journal/jkab433.

Chakraborty, A., Mahajan, S., Jaiswal, S.K., and Sharma, V.K. (2021). Genome sequencing of turmeric provides evolutionary insights into its medicinal properties. *Commun Biol* 4(1)**,** 1193. doi: 10.1038/s42003-021-02720-y.

Chen, D.X., Pan, Y., Wang, Y., Cui, Y.Z., Zhang, Y.J., Mo, R.Y., et al. (2021). The chromosome-level reference genome of *Coptis chinensis* provides insights into genomic evolution and berberine biosynthesis. *Hortic Res* 8(1)**,** 121. doi: 10.1038/s41438-021-00559-2.

Chen, H., Guo, M., Dong, S., Wu, X., Zhang, G., He, L., et al. (2023). A chromosome-scale genome assembly of *Artemisia argyi* reveals unbiased subgenome evolution and key contributions of gene duplication to volatile terpenoid diversity. *Plant Commun***,** 100516. doi: 10.1016/j.xplc.2023.100516.

Chen, Y., Fang, T., Su, H., Duan, S., Ma, R., Wang, P., et al. (2022). A reference-grade genome assembly for *Astragalus mongholicus* and insights into the biosynthesis and high accumulation of triterpenoids and flavonoids in its roots. *Plant Commun***,** 100469. doi: 10.1016/j.xplc.2022.100469.

Cheng, J., Wang, X., Liu, X., Zhu, X., Li, Z., Chu, H., et al. (2021). Chromosome-level genome of Himalayan yew provides insights into the origin and evolution of the paclitaxel biosynthetic pathway. *Mol Plant* 14(7)**,** 1199-1209. doi: 10.1016/j.molp.2021.04.015.

Cuello, C., Stander, E.A., Jansen, H.J., Dugé de Bernonville, T., Lanoue, A., Giglioli-Guivarc'h, N., et al. (2022). Genome assembly of the medicinal plant *Voacanga thouarsii*. *Genome Biol Evol* 14(11)**,** evac158. doi: 10.1093/gbe/evac158.

Cui, X., Meng, F., Pan, X., Qiu, X., Zhang, S., Li, C., et al. (2022). Chromosome-level genome assembly of *Aristolochia contorta* provides insights into the biosynthesis of benzylisoquinoline alkaloids and aristolochic acids. *Hortic Res* 9**,** uhac005. doi: 10.1093/hr/uhac005.

Davik, J., Røen, D., Lysøe, E., Buti, M., Rossman, S., Alsheikh, M., et al. (2022). A chromosome-level genome sequence assembly of the red raspberry (*Rubus idaeus* L.). *PLoS One* 17(3)**,** e0265096. doi: 10.1371/journal.pone.0265096.

Ding, X., Mei, W., Lin, Q., Wang, H., Wang, J., Peng, S., et al. (2020). Genome sequence of the agarwood tree *Aquilaria sinensis* (Lour.) Spreng: the first chromosome-level draft genome in the Thymelaeceae family. *Gigascience* 9(3)**,** giaa013. doi: 10.1093/gigascience/giaa013.

Dong, S., Liu, M., Liu, Y., Chen, F., Yang, T., Chen, L., et al. (2021). The genome of *Magnolia biondii* Pamp. provides insights into the evolution of Magnoliales and biosynthesis of terpenoids. *Hortic Res* 8(1)**,** 38. doi: 10.1038/s41438-021-00471-9.

Fan, G., Liu, X., Sun, S., Shi, C., Du, X., Han, K., et al. (2020). The chromosome level genome and genome-wide association study for the agronomic traits of *Panax notoginseng*. *iScience* 23(9)**,** 101538. doi: 10.1016/j.isci.2020.101538.

Feng, C., Feng, C., Lin, X., Liu, S., Li, Y., and Kang, M. (2021). A chromosome-level genome assembly provides insights into ascorbic acid accumulation and fruit softening in guava (*Psidium guajava*). *Plant Biotechnol J* 19(4)**,** 717-730. doi: 10.1111/pbi.13498.

Fu, A., Zheng, Y., Guo, J., Grierson, D., Zhao, X., Wen, C., et al. (2023). Telomere-to-telomere genome assembly of bitter melon (*Momordica charantia* L. var. *abbreviata* Ser.) reveals fruit development, composition and ripening genetic characteristics. *Hortic Res* 10(1)**,** uhac228. doi: 10.1093/hr/uhac228.

Fu, J., Wan, L., Song, L., He, L., Jiang, N., Long, H., et al. (2022). Chromosome-level genome assembly of the hemiparasitic *Taxillus chinensis* (DC.) Danser. *Genome Biol Evol* 14(5)**,** evac060. doi: 10.1093/gbe/evac060.

Gao, S., Wang, B., Xie, S., Xu, X., Zhang, J., Pei, L., et al. (2020). A high-quality reference genome of wild *Cannabis sativa*. *Hortic Res* 7(1)**,** 73. doi: 10.1038/s41438-020-0295-3.

Guo, C., Wang, Y., Yang, A., He, J., Xiao, C., Lv, S., et al. (2020). The *Coix* genome provides insights into Panicoideae evolution and papery hull domestication. *Mol Plant* 13(2)**,** 309-320. doi: 10.1016/j.molp.2019.11.008.

Guo, L., Winzer, T., Yang, X., Li, Y., Ning, Z., He, Z., et al. (2018). The opium poppy genome and morphinan production. *Science* 362(6412)**,** 343-347. doi: 10.1126/science.aat4096.

Han, B., Jing, Y., Dai, J., Zheng, T., Gu, F., Zhao, Q., et al. (2020). A chromosome-level genome assembly of *Dendrobium huoshanense* using long reads and Hi-C data. *Genome Biol Evol* 12(12)**,** 2486-2490. doi: 10.1093/gbe/evaa215.

Han, X., Li, C., Sun, S., Ji, J., Nie, B., Maker, G., et al. (2022). The chromosome-level genome of female ginseng (*Angelica sinensis*) provides insights into molecular mechanisms and evolution of coumarin biosynthesis. *Plant J* 112(5)**,** 1224-1237. doi: 10.1111/tpj.16007.

Hong, Z., Li, J., Liu, X., Lian, J., Zhang, N., Yang, Z., et al. (2020). The chromosome-level draft genome of *Dalbergia odorifera*. *Gigascience* 9(8)**,** giaa084. doi: 10.1093/gigascience/giaa084.

Hu, S., Wang, D., Wang, W., Zhang, C., Li, Y., Wang, Y., et al. (2022). Whole genome and transcriptome reveal flavone accumulation in *Scutellaria baicalensis* roots. *Front Plant Sci* 13**,** 1000469. doi: 10.3389/fpls.2022.1000469.

Hu, Y., Ma, D., Ning, S., Ye, Q., Zhao, X., Ding, Q., et al. (2021). High-quality genome of the medicinal plant *Strobilanthes cusia* provides insights into the biosynthesis of indole alkaloids. *Front Plant Sci* 12**,** 742420. doi: 10.3389/fpls.2021.742420.

Huang, H., Liang, J., Tan, Q., Ou, L., Li, X., Zhong, C., et al. (2021). Insights into triterpene synthesis and unsaturated fatty-acid accumulation provided by chromosomal-level genome analysis of *Akebia trifoliata* subsp. *australis*. *Hortic Res* 8(1)**,** 33. doi: 10.1038/s41438-020-00458-y.

Jaiswal, S.K., Mahajan, S., Chakraborty, A., Kumar, S., and Sharma, V.K. (2021). The genome sequence of *Aloe vera* reveals adaptive evolution of drought tolerance mechanisms. *iScience* 24(2)**,** 102079. doi: 10.1016/j.isci.2021.102079.

Jiang, L., Lin, M., Wang, H., Song, H., Zhang, L., Huang, Q., et al. (2022). Haplotype-resolved genome assembly of *Bletilla striata* (Thunb.) Reichb.f. to elucidate medicinal value. *Plant J* 111(5)**,** 1340-1353. doi: 10.1111/tpj.15892.

Jiang, S., An, H., Xu, F., and Zhang, X. (2020). Chromosome-level genome assembly and annotation of the loquat (*Eriobotrya japonica*) genome. *Gigascience* 9(3)**,** giaa015. doi: 10.1093/gigascience/giaa015.

Jiang, Z., Tu, L., Yang, W., Zhang, Y., Hu, T., Ma, B., et al. (2021). The chromosome-level reference genome assembly for *Panax notoginseng* and insights into ginsenoside biosynthesis. *Plant Commun* 2(1)**,** 100113. doi: 10.1016/j.xplc.2020.100113.

Kang, M., Fu, R., Zhang, P., Lou, S., Yang, X., Chen, Y., et al. (2021). A chromosome-level *Camptotheca acuminata* genome assembly provides insights into the evolutionary origin of camptothecin biosynthesis. *Nat Commun* 12(1)**,** 3531. doi: 10.1038/s41467-021-23872-9.

Kang, M., Wu, H., Yang, Q., Huang, L., Hu, Q., Ma, T., et al. (2020a). A chromosome-scale genome assembly of *Isatis indigotica*, an important medicinal plant used in traditional Chinese medicine: An *Isatis* genome. *Hortic Res* 7**,** 18. doi: 10.1038/s41438-020-0240-5.

Kang, S.H., Kim, B., Choi, B.S., Lee, H.O., Kim, N.H., Lee, S.J., et al. (2020b). Genome assembly and annotation of soft-shelled adlay (*Coix lacryma-jobi* variety *ma-yuen*), a cereal and medicinal crop in the Poaceae family. *Front Plant Sci* 11**,** 630. doi: 10.3389/fpls.2020.00630.

Kang, S.H., Pandey, R.P., Lee, C.M., Sim, J.S., Jeong, J.T., Choi, B.S., et al. (2020c). Genome-enabled discovery of anthraquinone biosynthesis in *Senna tora*. *Nat Commun* 11(1)**,** 5875. doi: 10.1038/s41467-020-19681-1.

Kaul, T., Easwaran, M., Thangaraj, A., Meyyazhagan, A., Nehra, M., Raman, N.M., et al. (2022). De novo genome assembly of rice bean (*Vigna umbellata*) - A nominated nutritionally rich future crop reveals novel insights into flowering potential, habit, and palatability centric - traits for efficient domestication. *Front Plant Sci* 13**,** 739654. doi: 10.3389/fpls.2022.739654.

Li, C.Y., Yang, L., Liu, Y., Xu, Z.G., Gao, J., Huang, Y.B., et al. (2022a). The sage genome provides insight into the evolutionary dynamics of diterpene biosynthesis gene cluster in plants. *Cell Rep* 40(7)**,** 111236. doi: 10.1016/j.celrep.2022.111236.

Li, H.L., Wu, L., Dong, Z., Jiang, Y., Jiang, S., Xing, H., et al. (2021). Haplotype-resolved genome of diploid ginger (*Zingiber officinale*) and its unique gingerol biosynthetic pathway. *Hortic Res* 8(1)**,** 189. doi: 10.1038/s41438-021-00627-7.

Li, L.F., Cushman, S.A., He, Y.X., and Li, Y. (2020a). Genome sequencing and population genomics modeling provide insights into the local adaptation of weeping *forsythia*. *Hortic Res* 7**,** 130. doi: 10.1038/s41438-020-00352-7.

Li, T., Yu, X., Ren, Y., Kang, M., Yang, W., Feng, L., et al. (2022b). The chromosome-level genome assembly of *Gentiana dahurica* (Gentianaceae) provides insights into gentiopicroside biosynthesis. *DNA Res* 29(2)**,** dsac008. doi: 10.1093/dnares/dsac008.

Li, Y., Wei, H., Yang, J., Du, K., Li, J., Zhang, Y., et al. (2020b). High-quality de novo assembly of the *Eucommia ulmoides* haploid genome provides new insights into evolution and rubber biosynthesis. *Hortic Res* 7(1)**,** 183. doi: 10.1038/s41438-020-00406-w.

Liang, Y., Chen, S., Wei, K., Yang, Z., Duan, S., Du, Y., et al. (2020). Chromosome level genome assembly of *Andrographis paniculata*. *Front Genet* 11**,** 701. doi: 10.3389/fgene.2020.00701.

Liao, B., Shen, X., Xiang, L., Guo, S., Chen, S., Meng, Y., et al. (2022). Allele-aware chromosome-level genome assembly of *Artemisia annua* reveals the correlation between ADS expansion and artemisinin yield. *Mol Plant* 15(8)**,** 1310-1328. doi: 10.1016/j.molp.2022.05.013.

Lin, M., Jian, J.B., Zhou, Z.Q., Chen, C.H., Wang, W., Xiong, H., et al. (2022). Chromosome-level genome of *Entada phaseoloides* provides insights into genome evolution and biosynthesis of triterpenoid saponins. *Mol Ecol Resour* 22(8)**,** 3049-3067. doi: 10.1111/1755-0998.13662.

Liu, H., Shi, J., Cai, Z., Huang, Y., Lv, M., Du, H., et al. (2020). Evolution and domestication footprints uncovered from the genomes of *Coix*. *Mol Plant* 13(2)**,** 295-308. doi: 10.1016/j.molp.2019.11.009.

Liu, Y., Wang, B., Shu, S., Li, Z., Song, C., Liu, D., et al. (2021). Analysis of the *Coptis chinensis* genome reveals the diversification of protoberberine-type alkaloids. *Nat Commun* 12(1)**,** 3276. doi: 10.1038/s41467-021-23611-0.

Luo, X., Li, H., Wu, Z., Yao, W., Zhao, P., Cao, D., et al. (2020). The pomegranate (*Punica granatum* L.) draft genome dissects genetic divergence between soft- and hard-seeded cultivars. *Plant Biotechnol J* 18(4)**,** 955-968. doi: 10.1111/pbi.13260.

Lv, S., Cheng, S., Wang, Z., Li, S., Jin, X., Lan, L., et al. (2020). Draft genome of the famous ornamental plant *Paeonia suffruticosa*. *Ecol Evol* 10(11)**,** 4518-4530. doi: 10.1002/ece3.5965.

Ma, L., Dong, C., Song, C., Wang, X., Zheng, X., Niu, Y., et al. (2021). De novo genome assembly of the potent medicinal plant *Rehmannia glutinosa* using nanopore technology. *Comput Struct Biotechnol J* 19**,** 3954-3963. doi: 10.1016/j.csbj.2021.07.006.

Ma, Q., Sun, T., Li, S., Wen, J., Zhu, L., Yin, T., et al. (2020). The *Acer truncatum* genome provides insights into nervonic acid biosynthesis. *Plant J* 104(3)**,** 662-678. doi: 10.1111/tpj.14954.

Matsumura, H., Hsiao, M.C., Lin, Y.P., Toyoda, A., Taniai, N., Tarora, K., et al. (2020). Long-read bitter gourd (*Momordica charantia*) genome and the genomic architecture of nonclassic domestication. *Proc Natl Acad Sci U S A* 117(25)**,** 14543-14551. doi: 10.1073/pnas.1921016117.

Miao, Y., Luo, D., Zhao, T., Du, H., Liu, Z., Xu, Z., et al. (2022). Genome sequencing reveals chromosome fusion and extensive expansion of genes related to secondary metabolism in *Artemisia argyi*. *Plant Biotechnol J* 20(10)**,** 1902-1915. doi: 10.1111/pbi.13870.

Mochida, K., Sakurai, T., Seki, H., Yoshida, T., Takahagi, K., Sawai, S., et al. (2017). Draft genome assembly and annotation of *Glycyrrhiza uralensis*, a medicinal legume. *Plant J* 89(2)**,** 181-194. doi: 10.1111/tpj.13385.

Niu, Z., Zhu, F., Fan, Y., Li, C., Zhang, B., Zhu, S., et al. (2021). The chromosome-level reference genome assembly for *Dendrobium officinale* and its utility of functional genomics research and molecular breeding study. *Acta Pharm Sin B* 11(7)**,** 2080-2092. doi: 10.1016/j.apsb.2021.01.019.

Peng, Z., Bredeson, J.V., Wu, G.A., Shu, S., Rawat, N., Du, D., et al. (2020). A chromosome-scale reference genome of trifoliate orange (*Poncirus trifoliata*) provides insights into disease resistance, cold tolerance and genome evolution in *Citrus*. *Plant J* 104(5)**,** 1215-1232. doi: 10.1111/tpj.14993.

Pootakham, W., Sonthirod, C., Naktang, C., Nawae, W., Yoocha, T., Kongkachana, W., et al. (2021). De novo assemblies of *Luffa acutangula* and *Luffa cylindrica* genomes reveal an expansion associated with substantial accumulation of transposable elements. *Mol Ecol Resour* 21(1)**,** 212-225. doi: 10.1111/1755-0998.13240.

Pu, X., Li, Z., Tian, Y., Gao, R., Hao, L., Hu, Y., et al. (2020). The honeysuckle genome provides insight into the molecular mechanism of carotenoid metabolism underlying dynamic flower coloration. *New Phytol* 227(3)**,** 930-943. doi: 10.1111/nph.16552.

Qin, S., Wu, L., Wei, K., Liang, Y., Song, Z., Zhou, X., et al. (2019). A draft genome for *Spatholobus suberectus*. *Sci Data* 6(1)**,** 113. doi: 10.1038/s41597-019-0110-x.

Rai, A., Hirakawa, H., Nakabayashi, R., Kikuchi, S., Hayashi, K., Rai, M., et al. (2021). Chromosome-level genome assembly of *Ophiorrhiza pumila* reveals the evolution of camptothecin biosynthesis. *Nat Commun* 12(1)**,** 405. doi: 10.1038/s41467-020-20508-2.

Rai, A., Hirakawa, H., Rai, M., Shimizu, Y., Shirasawa, K., Kikuchi, S., et al. (2022). Chromosome-scale genome assembly of *Glycyrrhiza uralensis* revealed metabolic gene cluster centred specialized metabolites biosynthesis. *DNA Res* 29(6)**,** dsac043. doi: 10.1093/dnares/dsac043.

Rajewski, A., Carter-House, D., Stajich, J., and Litt, A. (2021). *Datura* genome reveals duplications of psychoactive alkaloid biosynthetic genes and high mutation rate following tissue culture. *BMC Genomics* 22(1)**,** 201. doi: 10.1186/s12864-021-07489-2.

Roopa Sowjanya, P., Shilpa, P., Patil, G.P., Babu, D.K., Sharma, J., Sangnure, V.R., et al. (2022). Reference quality genome sequence of Indian pomegranate cv. 'Bhagawa' (*Punica granatum* L.). *Front Plant Sci* 13**,** 947164. doi: 10.3389/fpls.2022.947164.

Shen, G., Luo, Y., Yao, Y., Meng, G., Zhang, Y., Wang, Y., et al. (2022a). The discovery of a key prenyltransferase gene assisted by a chromosome-level *Epimedium pubescens* genome. *Front Plant Sci* 13**,** 1034943. doi: 10.3389/fpls.2022.1034943.

Shen, Q., Zhang, L., Liao, Z., Wang, S., Yan, T., Shi, P., et al. (2018). The genome of *Artemisia annua* provides insight into the evolution of Asteraceae family and artemisinin biosynthesis. *Mol Plant* 11(6)**,** 776-788. doi: 10.1016/j.molp.2018.03.015.

Shen, Y., Li, W., Zeng, Y., Li, Z., Chen, Y., Zhang, J., et al. (2022b). Chromosome-level and haplotype-resolved genome provides insight into the tetraploid hybrid origin of patchouli. *Nat Commun* 13(1)**,** 3511. doi: 10.1038/s41467-022-31121-w.

Song, C., Liu, Y., Song, A., Dong, G., Zhao, H., Sun, W., et al. (2018). The *Chrysanthemum nankingense* genome provides insights into the evolution and diversification of *Chrysanthemum* flowers and medicinal traits. *Mol Plant* 11(12)**,** 1482-1491. doi: 10.1016/j.molp.2018.10.003.

Song, X., Sun, P., Yuan, J., Gong, K., Li, N., Meng, F., et al. (2021). The celery genome sequence reveals sequential paleo-polyploidizations, karyotype evolution and resistance gene reduction in apiales. *Plant Biotechnol J* 19(4)**,** 731-744. doi: 10.1111/pbi.13499.

Song, Z., Lin, C., Xing, P., Fen, Y., Jin, H., Zhou, C., et al. (2020). A high-quality reference genome sequence of *Salvia miltiorrhiza* provides insights into tanshinone synthesis in its red rhizomes. *Plant Genome* 13(3)**,** e20041. doi: 10.1002/tpg2.20041.

Su, W., Jing, Y., Lin, S., Yue, Z., Yang, X., Xu, J., et al. (2021). Polyploidy underlies co-option and diversification of biosynthetic triterpene pathways in the apple tribe. *Proc Natl Acad Sci U S A* 118(20)**,** e2101767118. doi: 10.1073/pnas.2101767118.

Sun, F., Yan, C., Lv, Y., Pu, Z., Liao, Z., Guo, W., et al. (2022). Genome sequencing of *Amomum tsao-ko* provides novel insight into its volatile component biosynthesis. *Front Plant Sci* 13**,** 904178. doi: 10.3389/fpls.2022.904178.

Sun, G., Xu, Y., Liu, H., Sun, T., Zhang, J., Hettenhausen, C., et al. (2018). Large-scale gene losses underlie the genome evolution of parasitic plant *Cuscuta australis*. *Nat Commun* 9(1)**,** 2683. doi: 10.1038/s41467-018-04721-8.

Sun, W., Leng, L., Yin, Q., Xu, M., Huang, M., Xu, Z., et al. (2019). The genome of the medicinal plant *Andrographis paniculata* provides insight into the biosynthesis of the bioactive diterpenoid neoandrographolide. *Plant J* 97(5)**,** 841-857. doi: 10.1111/tpj.14162.

Taheri, S., Teo, C.H., Heslop-Harrison, J.S., Schwarzacher, T., Tan, Y.S., Wee, W.Y., et al. (2022). Genome assembly and analysis of the flavonoid and phenylpropanoid biosynthetic pathways in fingerroot ginger (*Boesenbergia rotunda*). *Int J Mol Sci* 23(13)**,** 7269. doi: 10.3390/ijms23137269.

Tamura, K., Sakamoto, M., Tanizawa, Y., Mochizuki, T., Matsushita, S., Kato, Y., et al. (2023). A highly contiguous genome assembly of red perilla (*Perilla frutescens*) domesticated in Japan. *DNA Res* 30(1). doi: 10.1093/dnares/dsac044.

Tu, L., Su, P., Zhang, Z., Gao, L., Wang, J., Hu, T., et al. (2020). Genome of *Tripterygium wilfordii* and identification of cytochrome P450 involved in triptolide biosynthesis. *Nat Commun* 11(1)**,** 971. doi: 10.1038/s41467-020-14776-1.

Usha, T., Middha, S.K., Babu, D., Goyal, A.K., Das, A.J., Saini, D., et al. (2022). Hybrid assembly and annotation of the genome of the Indian *Punica granatum*, a Superfood. *Front Genet* 13**,** 786825. doi: 10.3389/fgene.2022.786825.

Vadakkemukadiyil Chellappan, B., Pr, S., Vijayan, S., Rajan, V.S., Sasi, A., and Nair, A.S. (2019). High quality draft genome of arogyapacha (*Trichopus zeylanicus*), an important medicinal plant endemic to western ghats of India. *G3 (Bethesda)* 9(8)**,** 2395-2404. doi: 10.1534/g3.119.400164.

van Lieshout, N., van Kaauwen, M., Kodde, L., Arens, P., Smulders, M.J.M., Visser, R.G.F., et al. (2022). De novo whole-genome assembly of C*hrysanthemum makinoi*, a key wild chrysanthemum. *G3 (Bethesda)* 12(1)**,** jkab358. doi: 10.1093/g3journal/jkab358.

VanBuren, R., Wai, C.M., Colle, M., Wang, J., Sullivan, S., Bushakra, J.M., et al. (2018). A near complete, chromosome-scale assembly of the black raspberry (*Rubus occidentalis*) genome. *Gigascience* 7(8)**,** giy094. doi: 10.1093/gigascience/giy094.

Vining, K.J., Johnson, S.R., Ahkami, A., Lange, I., Parrish, A.N., Trapp, S.C., et al. (2017). Draft genome sequence of *Mentha longifolia* and development of resources for mint cultivar improvement. *Mol Plant* 10(2)**,** 323-339. doi: 10.1016/j.molp.2016.10.018.

Wang, F., Zhang, B., Wen, D., Liu, R., Yao, X., Chen, Z., et al. (2022a). Chromosome-scale genome assembly of *Camellia sinensis* combined with multi-omics provides insights into its responses to infestation with green leafhoppers. *Front Plant Sci* 13**,** 1004387. doi: 10.3389/fpls.2022.1004387.

Wang, J., Xie, J., Chen, H., Qiu, X., Cui, H., Liu, Y., et al. (2022b). A draft genome of the medicinal plant *Cremastra appendiculata* (D. Don) provides insights into the colchicine biosynthetic pathway. *Commun Biol* 5(1)**,** 1294. doi: 10.1038/s42003-022-04229-4.

Wang, J., Xu, S., Mei, Y., Cai, S., Gu, Y., Sun, M., et al. (2021a). A high-quality genome assembly of *Morinda officinalis*, a famous native southern herb in the Lingnan region of southern China. *Hortic Res* 8(1)**,** 135. doi: 10.1038/s41438-021-00551-w.

Wang, M., Gu, Z., Fu, Z., and Jiang, D. (2021b). High-quality genome assembly of an important biodiesel plant, *Euphorbia lathyris* L. *DNA Res* 28(6)**,** dsab022. doi: 10.1093/dnares/dsab022.

Wang, X., Xu, Y., Zhang, S., Cao, L., Huang, Y., Cheng, J., et al. (2017). Genomic analyses of primitive, wild and cultivated citrus provide insights into asexual reproduction. *Nat Genet* 49(5)**,** 765-772. doi: 10.1038/ng.3839.

Wang, X.D., Xu, C.Y., Zheng, Y.J., Wu, Y.F., Zhang, Y.T., Zhang, T., et al. (2022c). Chromosome-level genome assembly and resequencing of camphor tree (*Cinnamomum camphora*) provides insight into phylogeny and diversification of terpenoid and triglyceride biosynthesis of *Cinnamomum*. *Hortic Res* 9**,** uhac216. doi: 10.1093/hr/uhac216.

Wang, Y., and Shahid, M.Q. (2022). Genome sequencing and resequencing identified three horizontal gene transfers and uncovered the genetic mechanism on the intraspecies adaptive evolution of *Gastrodia elata* Blume. *Front Plant Sci* 13**,** 1035157. doi: 10.3389/fpls.2022.1035157.

Wei, C., Yang, H., Wang, S., Zhao, J., Liu, C., Gao, L., et al. (2018). Draft genome sequence of *Camellia sinensis* var. *sinensis* provides insights into the evolution of the tea genome and tea quality. *Proc Natl Acad Sci U S A* 115(18)**,** E4151-e4158. doi: 10.1073/pnas.1719622115.

Wu, H., Zhao, G., Gong, H., Li, J., Luo, C., He, X., et al. (2020). A high-quality sponge gourd (*Luffa cylindrica*) genome. *Hortic Res* 7(1)**,** 128. doi: 10.1038/s41438-020-00350-9.

Wu, Z., Liu, H., Zhan, W., Yu, Z., Qin, E., Liu, S., et al. (2021). The chromosome-scale reference genome of safflower (*Carthamus tinctorius*) provides insights into linoleic acid and flavonoid biosynthesis. *Plant Biotechnol J* 19(9)**,** 1725-1742. doi: 10.1111/pbi.13586.

Wuyun, T.N., Wang, L., Liu, H., Wang, X., Zhang, L., Bennetzen, J.L., et al. (2018). The hardy rubber tree genome provides insights into the evolution of polyisoprene biosynthesis. *Mol Plant* 11(3)**,** 429-442. doi: 10.1016/j.molp.2017.11.014.

Xia, M., Han, X., He, H., Yu, R., Zhen, G., Jia, X., et al. (2018). Improved de novo genome assembly and analysis of the Chinese cucurbit *Siraitia grosvenorii*, also known as monk fruit or luo-han-guo. *Gigascience* 7(6). doi: 10.1093/gigascience/giy067.

Xian, L., Sahu, S.K., Huang, L., Fan, Y., Lin, J., Su, J., et al. (2022). The draft genome and multi-omics analyses reveal new insights into geo-herbalism properties of *Citrus grandis* 'Tomentosa'. *Plant Sci* 325**,** 111489. doi: 10.1016/j.plantsci.2022.111489.

Xiong, X., Gou, J., Liao, Q., Li, Y., Zhou, Q., Bi, G., et al. (2021). The *Taxus* genome provides insights into paclitaxel biosynthesis. *Nat Plants* 7(8)**,** 1026-1036. doi: 10.1038/s41477-021-00963-5.

Xu, H., Song, J., Luo, H., Zhang, Y., Li, Q., Zhu, Y., et al. (2016). Analysis of the genome sequence of the medicinal plant *Salvia miltiorrhiza*. *Mol Plant* 9(6)**,** 949-952. doi: 10.1016/j.molp.2016.03.010.

Xu, Q., Niu, S.C., Li, K.L., Zheng, P.J., Zhang, X.J., Jia, Y., et al. (2022a). Chromosome-scale assembly of the *Dendrobium nobile* genome provides insights into the molecular mechanism of the biosynthesis of the medicinal active ingredient of *Dendrobium*. *Front Genet* 13**,** 844622. doi: 10.3389/fgene.2022.844622.

Xu, S., Li, F., Wu, B., Mei, Y., Wang, J., and Wang, J. (2022b). Complete genome sequence and phylogenetic analysis of medicinal plant *Abrus cantoniensis* for evolutionary research and germplasm utilization. *Plant Genome* 15(3)**,** e20236. doi: 10.1002/tpg2.20236.

Xu, X., Yuan, H., Yu, X., Huang, S., Sun, Y., Zhang, T., et al. (2021). The chromosome-level *Stevia* genome provides insights into steviol glycoside biosynthesis. *Hortic Res* 8(1)**,** 129. doi: 10.1038/s41438-021-00565-4.

Xu, Y., Zhang, K., Zhang, Z., Liu, Y., Lv, F., Sun, P., et al. (2022c). A chromosome-level genome assembly for *Dracaena cochinchinensis* reveals the molecular basis of its longevity and formation of dragon's blood. *Plant Commun* 3(6)**,** 100456. doi: 10.1016/j.xplc.2022.100456.

Xu, Z., Gao, R., Pu, X., Xu, R., Wang, J., Zheng, S., et al. (2020a). Comparative genome analysis of *Scutellaria baicalensis* and *Scutellaria barbata* reveals the evolution of active flavonoid biosynthesis. *Genomics Proteomics Bioinformatics* 18(3)**,** 230-240. doi: 10.1016/j.gpb.2020.06.002.

Xu, Z., Li, Z., Ren, F., Gao, R., Wang, Z., Zhang, J., et al. (2022d). The genome of *Corydalis* reveals the evolution of benzylisoquinoline alkaloid biosynthesis in Ranunculales. *Plant J* 111(1)**,** 217-230. doi: 10.1111/tpj.15788.

Xu, Z., Pu, X., Gao, R., Demurtas, O.C., Fleck, S.J., Richter, M., et al. (2020b). Tandem gene duplications drive divergent evolution of caffeine and crocin biosynthetic pathways in plants. *BMC Biol* 18(1)**,** 63. doi: 10.1186/s12915-020-00795-3.

Xu, Z., Xin, T., Bartels, D., Li, Y., Gu, W., Yao, H., et al. (2018). Genome analysis of the ancient tracheophyte *Selaginella tamariscina* reveals evolutionary features relevant to the acquisition of desiccation tolerance. *Mol Plant* 11(7)**,** 983-994. doi: 10.1016/j.molp.2018.05.003.

Yan, L., Wang, X., Liu, H., Tian, Y., Lian, J., Yang, R., et al. (2015). The genome of *Dendrobium officinale* illuminates the biology of the important traditional Chinese orchid herb. *Mol Plant* 8(6)**,** 922-934. doi: 10.1016/j.molp.2014.12.011.

Yang, P., Zhao, H.Y., Wei, J.S., Zhao, Y.Y., Lin, X.J., Su, J., et al. (2022a). Chromosome-level genome assembly and functional characterization of terpene synthases provide insights into the volatile terpenoid biosynthesis of *Wurfbainia villosa*. *Plant J* 112(3)**,** 630-645. doi: 10.1111/tpj.15968.

Yang, Y., Huang, L., Xu, C., Qi, L., Wu, Z., Li, J., et al. (2021a). Chromosome-scale genome assembly of areca palm (*Areca catechu*). *Mol Ecol Resour* 21(7)**,** 2504-2519. doi: 10.1111/1755-0998.13446.

Yang, Y., Li, S., Xing, Y., Zhang, Z., Liu, T., Ao, W., et al. (2022b). The first high-quality chromosomal genome assembly of a medicinal and edible plant *Arctium lappa*. *Mol Ecol Resour* 22(4)**,** 1493-1507. doi: 10.1111/1755-0998.13547.

Yang, Y., Zhang, B., Bao, Y., Huang, P., Li, J., Li, R., et al. (2023). Chromosome-level genome assembly of *Herpetospermum pedunculosum* (Cucurbitaceae). *Genome Biol Evol*. doi: 10.1093/gbe/evad005.

Yang, Z., Chen, S., Wang, S., Hu, Y., Zhang, G., Dong, Y., et al. (2021b). Chromosomal-scale genome assembly of *Eleutherococcus senticosus* provides insights into chromosome evolution in Araliaceae. *Mol Ecol Resour* 21(7)**,** 2204-2220. doi: 10.1111/1755-0998.13403.

Ye, L., Yang, L., Wang, B., Chen, G., Jiang, L., Hu, Z., et al. (2022). The Chromosome-level genome of *Aesculus wilsonii* provides new insights into terpenoid biosynthesis and *Aesculus* evolution. *Front Plant Sci* 13**,** 1022169. doi: 10.3389/fpls.2022.1022169.

Yin, Y., Xie, X., Zhou, L., Yin, X., Guo, S., Zhou, X., et al. (2022). A chromosome-scale genome assembly of turmeric provides insights into curcumin biosynthesis and tuber formation mechanism. *Front Plant Sci* 13**,** 1003835. doi: 10.3389/fpls.2022.1003835.

Yu, H., Guo, K., Lai, K., Shah, M.A., Xu, Z., Cui, N., et al. (2022). Chromosome-scale genome assembly of an important medicinal plant honeysuckle. *Sci Data* 9(1)**,** 226. doi: 10.1038/s41597-022-01385-4.

Zhang, G., Tian, Y., Zhang, J., Shu, L., Yang, S., Wang, W., et al. (2015). Hybrid de novo genome assembly of the Chinese herbal plant danshen (*Salvia miltiorrhiza* Bunge). *Gigascience* 4**,** 62. doi: 10.1186/s13742-015-0104-3.

Zhang, L., Liu, M., Long, H., Dong, W., Pasha, A., Esteban, E., et al. (2019). Tung tree (*Vernicia fordii*) genome provides a resource for understanding genome evolution and improved oil production. *Genomics Proteomics Bioinformatics* 17(6)**,** 558-575. doi: 10.1016/j.gpb.2019.03.006.

Zhang, Q., Li, M., Chen, X., Liu, G., Zhang, Z., Tan, Q., et al. (2022a). Chromosome-level genome assembly of *Bupleurum chinense* DC provides insights into the saikosaponin biosynthesis. *Front Genet* 13**,** 878431. doi: 10.3389/fgene.2022.878431.

Zhang, T., Ren, X., Zhang, Z., Ming, Y., Yang, Z., Hu, J., et al. (2020). Long-read sequencing and de novo assembly of the *Luffa cylindrica* (L.) Roem. genome. *Mol Ecol Resour* 20(2)**,** 511-519. doi: 10.1111/1755-0998.13129.

Zhang, W., Xu, S., Gu, Y., Jiao, M., Mei, Y., and Wang, J. (2022b). The first high-quality chromosome-level genome assembly of Phyllanthaceae (*Phyllanthus cochinchinensis*) provides insights into flavonoid biosynthesis. *Planta* 256(6)**,** 109. doi: 10.1007/s00425-022-04026-7.

Zhang, X., Zhao, Y., Kou, Y., Chen, X., Yang, J., Zhang, H., et al. (2023). Diploid chromosome-level reference genome and population genomic analyses provide insights into Gypenoside biosynthesis and demographic evolution of *Gynostemma pentaphyllum* (Cucurbitaceae). *Hortic Res* 10(1)**,** uhac231. doi: 10.1093/hr/uhac231.

Zhao, Q., Yang, J., Cui, M.Y., Liu, J., Fang, Y., Yan, M., et al. (2019). The reference genome sequence of *Scutellaria baicalensis* provides insights into the evolution of wogonin biosynthesis. *Mol Plant* 12(7)**,** 935-950. doi: 10.1016/j.molp.2019.04.002.

Zheng, X., Chen, D., Chen, B., Liang, L., Huang, Z., Fan, W., et al. (2021). Insights into salvianolic acid B biosynthesis from chromosome-scale assembly of the *Salvia bowleyana* genome. *J Integr Plant Biol* 63(7)**,** 1309-1323. doi: 10.1111/jipb.13085.

Zhong, S., Li, B., Chen, W., Wang, L., Guan, J., Wang, Q., et al. (2022). The chromosome-level genome of *Akebia trifoliata* as an important resource to study plant evolution and environmental adaptation in the Cretaceous. *Plant J* 112(5)**,** 1316-1330. doi: 10.1111/tpj.16011.

Zhou, T., Bai, G., Hu, Y., Ruhsam, M., Yang, Y., and Zhao, Y. (2022a). De novo genome assembly of the medicinal plant *Gentiana macrophylla* provides insights into the genomic evolution and biosynthesis of iridoids. *DNA Res* 29(6)**,** dsac034. doi: 10.1093/dnares/dsac034.

Zhou, W., Wang, Y., Li, B., Petijová, L., Hu, S., Zhang, Q., et al. (2021). Whole-genome sequence data of *Hypericum perforatum* and functional characterization of melatonin biosynthesis by N-acetylserotonin O-methyltransferase. *J Pineal Res* 70(2)**,** e12709. doi: 10.1111/jpi.12709.

Zhou, Y., Fan, W., Zhang, H., Zhang, J., Zhang, G., Wang, D., et al. (2022b). The genome of *Marsdenia tenacissima* provides insights into calcium adaptation and tenacissoside biosynthesis. *Plant J*. doi: 10.1111/tpj.16081.
